# Ketamine’s rapid and sustained antidepressant effects are driven by distinct mechanisms

**DOI:** 10.1007/s00018-024-05121-6

**Published:** 2024-02-27

**Authors:** Radhika Rawat, Elif Tunc-Ozcan, Sara Dunlop, Yung-Hsu Tsai, Fangze Li, Ryan Bertossi, Chian-Yu Peng, John A. Kessler

**Affiliations:** Department of Neurology, Feinberg School of Medicine, Northwestern University, 303 E. Chicago Ave, Ward 10-233, Chicago, IL 60611 USA

**Keywords:** Ketamine, Antidepressant, Mechanism, Adult neurogenesis, BMP, Hippocampus

## Abstract

Administration of multiple subanesthetic doses of ketamine increases the duration of antidepressant effects relative to a single ketamine dose, but the mechanisms mediating this sustained effect are unclear. Here, we demonstrate that ketamine’s rapid and sustained effects on affective behavior are mediated by separate and temporally distinct mechanisms. The rapid effects of a single dose of ketamine result from increased activity of immature neurons in the hippocampal dentate gyrus without an increase in neurogenesis. Treatment with six doses of ketamine over two weeks doubled the duration of behavioral effects after the final ketamine injection. However, unlike ketamine’s rapid effects, this more sustained behavioral effect did not correlate with increased immature neuron activity but instead correlated with increased numbers of calretinin-positive and doublecortin-positive immature neurons. This increase in neurogenesis was associated with a decrease in bone morphogenetic protein (BMP) signaling, a known inhibitor of neurogenesis. Injection of a BMP4-expressing lentivirus into the dentate gyrus maintained BMP signaling in the niche and blocked the sustained – but not the rapid – behavioral effects of ketamine, indicating that decreased BMP signaling is necessary for ketamine’s sustained effects. Thus, although the rapid effects of ketamine result from increased activity of immature neurons in the dentate gyrus without requiring an increase in neurogenesis, ketamine’s sustained effects require a decrease in BMP signaling and increased neurogenesis along with increased neuron activity. Understanding ketamine’s dual mechanisms of action should help with the development of new rapid-acting therapies that also have safe, reliable, and sustained effects.

**Supplementary Information:**

The online version contains supplementary material available at 10.1007/s00018-024-05121-6.

## Introduction

Despite the breadth of current treatments for Major Depressive Disorder (MDD), there remains a large unmet clinical need for fast-acting effective therapeutics. As many as 1/3 of patients are resistant to current first-line antidepressants [1–3], and for those who do respond, symptomatic improvement is delayed for one to six weeks [4], a period during which patients experience an elevated risk for morbidity and mortality. By contrast, even in treatment-resistant patients, a single administration of low-dose ketamine can decrease depressive symptoms within two hours with effects that last approximately one week [5–7]. Patients who receive multiple ketamine doses experience a longer duration of antidepressant effects after the final administration [7–9]. For example, administration of 6 doses of ketamine over 12 days results in significant antidepressant effects that persist for 18–28 days after the final injection [7–9]. However, repeated ketamine administration carries significant risks including cardiovascular side effects and addiction [10–13]. These limitations highlight the need for mechanistic understanding to inform the development of safer antidepressants with both rapid and sustained effects.

Ketamine is a non-competitive N-methyl-D-aspartate (NMDA) receptor antagonist. However, not all NMDA receptor antagonists reproduce ketamine’s behavioral effects [14–17], implicating additional mechanisms of action [18–22]. Ketamine’s rapid effects require AMPA receptor activation, increased activity of immature neurons in the hippocampal dentate gyrus (DG), increased BDNF signaling, and synaptic changes in multiple regions of the brain [14, 23–26]. However, the acute and subacute alterations caused by a single dose are better studied than the effects of multiple doses, especially over time [27, 28], and it is unclear whether the mechanisms underlying ketamine’s rapid effects also underlie the more prolonged effects of repeat dosing.

Ketamine exerts effects on many regions of the brain, notably in the hippocampus [15, 21, 29–32]. Hippocampal dysfunction and volume loss are known hallmarks of MDD [33, 34]. Adult Hippocampal Neurogenesis (AHN) is also disrupted, and conventional antidepressant medications restore AHN [35–37]. Inhibiting AHN blocks the effects of antidepressant medications on behavioral phenotypes in pre-clinical models [35, 38–43]. Further, silencing adult-born immature neurons prevents the behavioral effects of antidepressants in mice, indicating a causal relationship between antidepressant-mediated changes in AHN and behavior [44]. Many different classes of antidepressants require AHN to affect behavior, suggesting convergent mechanisms of action.

One point of convergence for most major classes of antidepressants is the inhibitory effect of bone morphogenic protein (BMP) signaling in the hippocampus [45]. BMP signaling inhibits AHN and decreased BMP signaling is necessary for both the cellular and behavioral effects of many classes of conventional antidepressant medications [45–47]. In the present study, we show that the sustained behavioral effects of ketamine are mediated by a decrease in BMP signaling and an increase in AHN, without selectively altering the activity of immature neurons. By contrast, the rapid onset effects are ketamine have been shown to reflect the increased activity of immature neurons, without any change in AHN [27]. Thus, the rapid onset and sustained effects of ketamine are mediated by separate and temporally distinct mechanisms, and decreased BMP signaling is necessary for maintaining, but not initiating, ketamine’s behavioral effects. Kindly check the order of the section headings are correct and amend if necessary. correct

## Results

### The behavioral effects of multiple ketamine doses outlast the effects of a single dose

We first examined the magnitude and duration of behavioral effects after single or multiple intraperitoneal (i.p.) injections of subanesthetic ketamine (3 mg/kg) in 8–10-week-old male and female C57BL6 mice. We used three widely used tests of hippocampus-dependent stress behavior and social memory [48, 49] throughout this study: the social interaction (SIT), social novelty (SNT), and tail suspension (TST) tests [44, 47–51]. All experimental mice in this study underwent unpredictable chronic mild stress (UCMS) to induce robust stress- and anxiety-induced behavioral phenotype [44, 52–59].Please confirm the section headings are correctly identified.correct

**Fig. 1:**
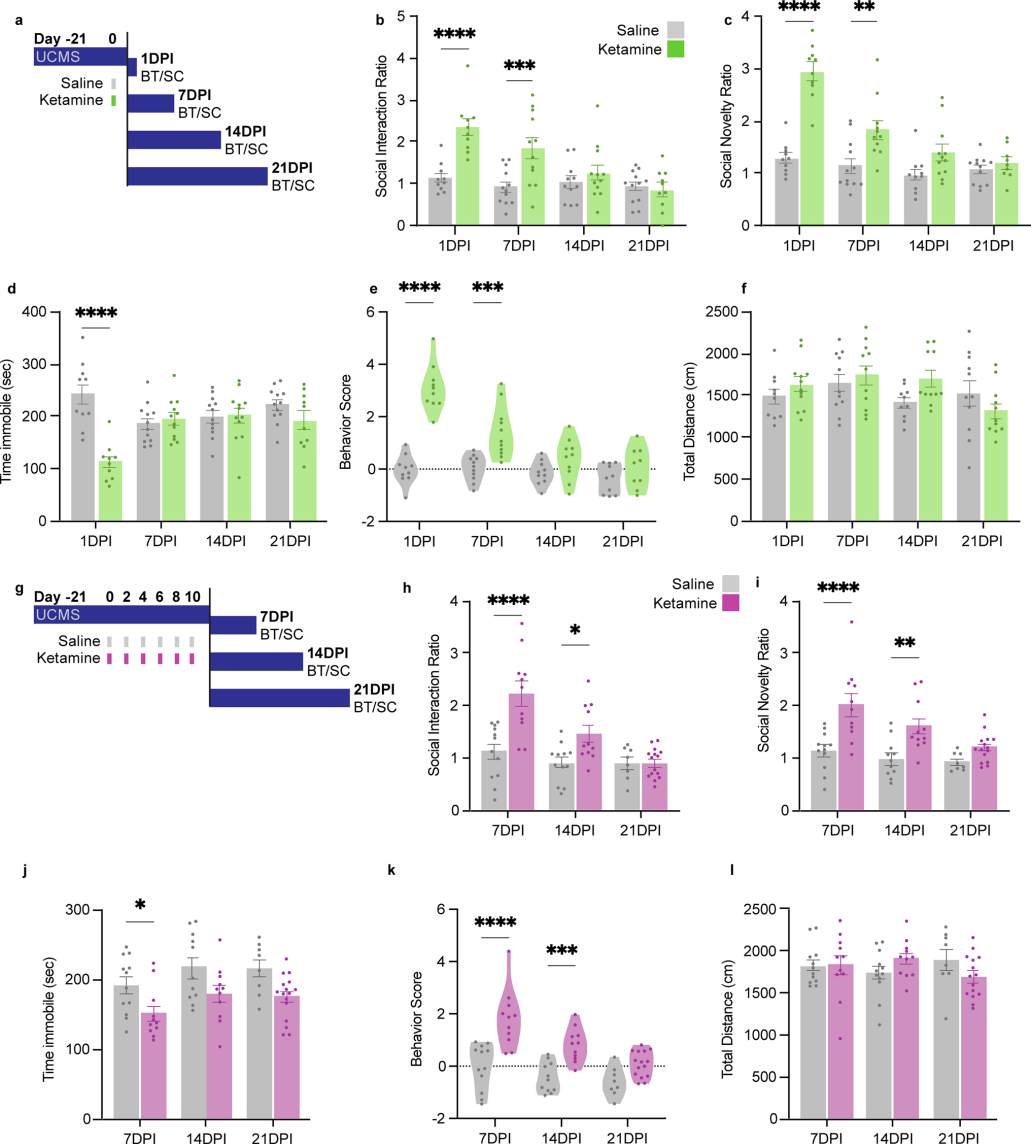
Multiple doses of ketamine increase and extend ketamine’s behavioral effects. **a** 8–10-week-old male and female mice underwent UCMS (dark blue bar) and were given a single dose of subanesthetic (3 mg/kg) ketamine (K; green) or saline (S; grey) by intraperitoneal injection mice prior to behavioral testing (BT) and sample collection (SC), either 1-, 7-, 14-, or 21-days post injection (DPI). **b** Ketamine-treated mice had higher Social Interaction Ratios (time spent interacting with an unfamiliar mouse divided by time spent interacting with an object in the Social Interaction Test, SIT) than saline-treated mice at 1DPI and 7DPI. Two-way ANOVA: Interaction **p = 0.0012, DPI ****p < 0.0001, ketamine ****p < 0.0001. Šídák's multiple comparisons test: 1DPI S (*n* = 10) v K (*n* = 10) ****p < 0.0001, 7DPI S (*n* = 12) v K (*n* = 12) ****p < 0.0001, 14DPI S (*n* = 11) v K (*n* = 12) *p < 0.05, 21DPI S (*n* = 12) v K (*n* = 10) ns *p* > 0.9999. **c** Ketamine-treated mice had higher Social Novelty Ratios (time spent interacting with an unfamiliar mouse divided by time spent interacting with a familiar mouse in the Social Novelty Test, SNT) at 1DPI and 7DPI. Two-way ANOVA: Interaction ****p < 0.0001, DPI ****p < 0.0001, Ketamine ****p < 0.0001. Šídák’s multiple comparisons test: 1DPI S (*n* = 10) v K (*n* = 10) ****p < 0.0001, 7DPI S (*n* = 12) v K (*n* = 11) **p < 0.0012, 14DPI S (*n* = 10) v K (*n* = 12) ns *p* < 0.1130, 21DPI S (*n* = 12) v K (*n* = 9) ns *p* = 0.9398. **d** Ketamine-treated mice spent less time immobile (sec) during the Tail Suspension Test (TST) than saline mice only at 1DPI. Two-way ANOVA: Interaction ****p < 0.0001, DPI *p* = 0.1424, Ketamine ****p = 0.0001. Šídák's multiple comparisons test: 1DPI S (*n* = 10) v K (*n* = 10) ****p < 0.0001, 7DPI S (*n* = 12) v K (*n* = 12) ns *p* = 0.9842, 14DPI S (*n* = 12) v K (*n* = 12) ns *p* > 0.9999, 21DPI S (*n* = 12) v K (*n* = 10) ns *p* = 0.4197. **e** Ketamine-treated mice had significantly higher behavior scores than saline-treated mice at 1DPI and 7DPI. Two-way ANOVA: Interaction ****p < 0.0001, DPI ****p < 0.0001, Ketamine ****p < 0.0001. Šídák's multiple comparisons test: 1DPI S (*n* = 10) v K (*n* = 10) ns *p* < 0.0001, 7DPI S (*n* = 11) v K (*n* = 11) *p* = 0.0001, 14DPI S (*n* = 10) v K (*n* = 10) ns *p* = 0.3593, 21DPI S (*n* = 10) v K (*n* = 9) ns *p* = 0.5996. **f** There was no significant difference in locomotion as measured by the Total Distance travelled (cm) in the Open Field Test (OFT). Two-way ANOVA: Interaction *p* = 0.1141, DPI *p* = 0.0765, Ketamine *p* = 0.3025. Šídák's multiple comparisons test: 1DPI S (*n* = 10) v K (*n* = 12) ns *p* = 0.7994, 7DPI S (*n* = 11) v K (*n* = 11) *p* = 0.9482, 14DPI S (*n* = 10) v K (*n* = 11) ns *p* = 0.2246, 21DPI S (*n* = 11) v K (*n* = 11) ns *p* = 0.4627. **g** 8–10-week-old male and female mice underwent unpredictable chronic mild stress (UCMS) and received 6 doses of subanesthetic (3 mg/kg) ketamine (K; purple) or saline (S; grey) by intraperitoneal injection prior to BT and SC either 7, 14, or 21 days after the final injection (DPI). **h** Mice that received multiple ketamine doses had higher Social Interaction Ratios than saline-treated mice at 7DPI. Two-way ANOVA: Interaction **p = 0.0020, DPI ***p < 0.0001, ketamine ****p < 0.0001; Šídák's multiple comparisons test, S (*n* = 12) v K (*n* = 11): 7DPI ****p < 0.0001, S (*n* = 12) v K (*n* = 11): 14DPI *p < 0.05, S (*n* = 8) v K (*n* = 15): 21DPI ns *p* > 0.9999. **i** Mice that received multiple ketamine doses had higher Social Novelty Ratios than saline-treated mice at 7DPI and 14DPI. Two-way ANOVA: Interaction ns *p* = 0.0845, DPI **p = 0.0012, ketamine ****p < 0.0001; Šídák's multiple comparisons test, S (*n* = 12) v K (*n* = 11): 7DPI ****p < 0.0001, S (*n* = 11) v K (*n* = 11): 14DPI **p = 0.0049, S (*n* = 8) v K (*n* = 15): 21DPI ns *p* = 0.4086. **j** Mice that received multiple ketamine doses spent less time immobile (sec) during the TST at 7DPI. Two-way ANOVA: Interaction ns *p* = 0.9846, DPI *p = 0.0446, ketamine ***p = 0.0001; Šídák's multiple comparisons test, S (*n* = 12) v K (*n* = 11): 7DPI *p < 0.0442, S (*n* = 11) v K (*n* = 11): 14DPI ns *p* = 0.0859, S (*n* = 8) v K (*n* = 16): 21DPI ns *p* = 0.0572. **k** Mice that received multiple ketamine doses had significantly higher behavior scores than saline-treated mice at both 7DPI and 14DPI. Two-way ANOVA: Interaction: *p = 0.0469, DPI ****p < 0.0001, Ketamine ****p < 0.0001. Šídák's multiple comparisons test, S (*n* = 12) v K (*n* = 11): 7DPI ****p < 0.0001, S (*n* = 11) v K (*n* = 11): 14DPI ***p = 0.0005, S (*n* = 8) v K (*n* = 15): 21DPI ns *p* = 0.1039. **l** There were no differences in locomotion, as measured by the total distance travelled in the OFT, between mice that received multiple doses of ketamine or multiple doses of saline. Two-way ANOVA: Interaction ns *p* = 0.0845, DPI **p = 0.0012, ketamine ****p < 0.0001; Šídák's multiple comparisons test, S (*n* = 12) v K (*n* = 11): 7DPI ****p < 0.0001, S (*n* = 11) v K (*n* = 11): 14DPI **p = 0.0049, S (*n* = 8) v K (*n* = 15): 21DPI ns *p* = 0.4086

After three weeks of UCMS as previously described [26, 44], mice received one dose of saline or ketamine, and then underwent behavioral testing (BT) and sample collection (SC) either at 1-, 7-, 14-, or 21-days post injection (DPI) (Fig. 1a). A single subanesthetic dose of ketamine produced relatively short-lived effects. Compared to saline-treated mice, single-dose ketamine-treated mice were more sociable in the SIT (Fig. 1b), measured by the Social Interaction Ratio, or time spent with an unfamiliar mouse vs an inanimate object, and had an increased preference for social novelty in the SNT, measured by the time spent with unfamiliar mouse vs a familiar one (Fig. 1c) at 1 and 7 DPI, but not at 14 or 21 DPI. There was no effect of ketamine on time immobile in the TST after 1 DPI (Fig. 1d). A combined behavior score was calculated for each mouse (see Methods) such that the z-scores of each behavioral output measure were averaged into a composite z score for each individual mouse [60, 61], and a positive score indicates a positive behavioral effect relative to the control group. After a single dose of ketamine, effects on behavior were significant only at 1 and 7 DPI (Fig. 1e). Ketamine did not affect locomotor activity, measured by total distance traveled in the Open Field Test (OFT, Fig. 1f). No significant behavioral differences were observed between males and females (Supp. Figure 1).To examine the effects following multiple ketamine doses, mice underwent 3 weeks of UCMS and then received 6 doses of ketamine (one dose every other day for 10 days). The mice underwent BT at 7, 14, and 21 days after the final ketamine injection (days post-injection, DPI) (Fig. 1g). Mice that received multiple doses of ketamine displayed more persistent behavioral effects than mice that received a single dose. Relative to saline-treated mice, ketamine-treated mice had significantly increased sociability (Fig. 1h) and preference for social novelty (Fig. 1i) at both 7 and 14 DPI, as well as significantly decreased time immobile on the TST at 7 DPI (Fig. 1j). After multiple doses of ketamine, the effect on the behavior score was significant at 7 and 14 DPI, with a trend towards significance at 21 DPI (Fig. 1k), indicating an increased duration of behavioral improvements after cessation of treatment compared to the single dose (Supp. Figure 1). The total distance traveled during the OFT remained constant over time (Fig. 1l). No significant behavioral differences were observed between males and females (Supp. Figure 1).

### The sustained behavioral effects of multiple doses of ketamine correlate with the number of immature neurons but not their activity

**Fig. 2:**
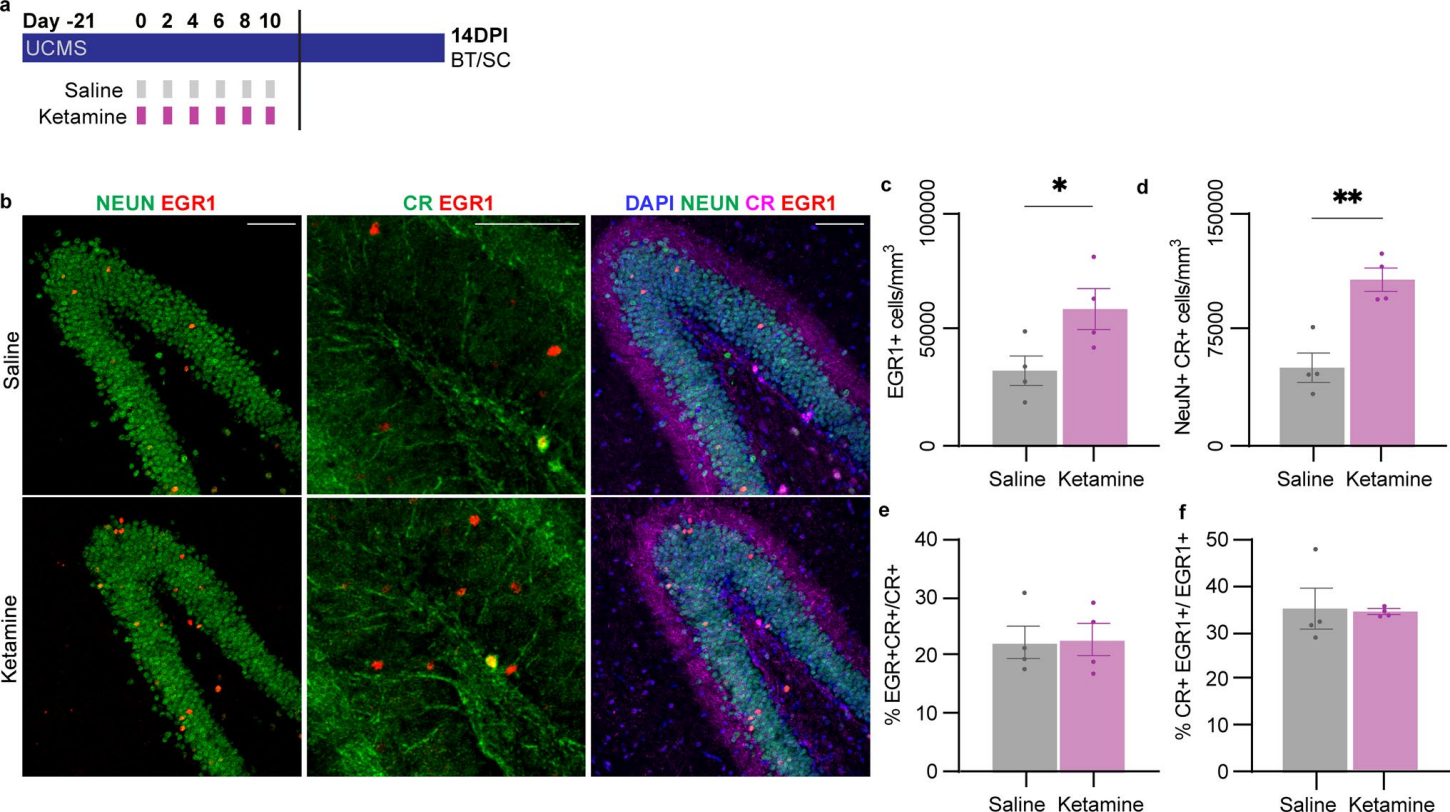
Multiple doses of ketamine do not increase the activity of immature neurons. **a** 8–10-week-old mice underwent unpredictable chronic mild stress (UCMS) and received 6 doses of subanesthetic (3 mg/kg) ketamine (K; purple) or saline (S; grey) by intraperitoneal injection prior to BT and SC for immunohistochemistry 14 days after the final injection (14DPI). **b** Representative fluorescent staining and colocalization of NeuN (green), EGR1 (red), CR (magenta), and DAPI (blue, nuclear stain) in the dentate gyrus (DG) of the hippocampus. Scale bars represent 50um. **c** EGR1 + cells/mm^3^ were higher in ketamine-treated mouse DGs than saline-treated mouse DGs (*n* = 4 mice/group). Unpaired, two-tailed *t* test: *p = 0.0491. **d** NeuN + CR + cells/mm^3^ were higher in ketamine-treated mouse DGs than saline-treated mouse DGs (*n* = 4 mice/group). Unpaired, two-tailed *t* test: **p = 0.0030. **e** The percentage of NeuN + CR + cells that were also EGR1 + was not different between ketamine- and saline-treated mouse DGs (*n* = 4 mice/group). Unpaired, two-tailed *t* test: *p* = 0.9296. **f** The percentage of EGR1 + cells that are also NeuN + CR + was not different between ketamine- and saline-treated mouse DGs (*n* = 4 mice/group). Unpaired, two-tailed *t* test: ns *p* = 0.8535. Data were presented as means ± s.e.m. Each replicate is a different mouse, with each point representing the average from 3 to 5 hippocampal sections per mouse. *p < 0.05, **p < 0.01, ***p < 0.001, ***p < 0.0001

Preference for social interaction, novelty, and several other stress-related, affective behaviors are regulated by the hippocampus and the DG [44, 47, 50, 51, 62–68]. We previously demonstrated that the rapid behavioral effects of ketamine require increased adult-born immature dentate granule neuron activity without a change in AHN or immature neuronal numbers [26], and we, therefore, asked whether the sustained antidepressant effects of multiple doses of ketamine are mediated by a similar mechanism. Fourteen days after the last saline or ketamine treatment in the multiple-dose regimen (Fig. 2a), we collected samples for immunohistochemistry (IHC) and quantified the number of cells labeled with the markers Early Growth Response 1 (EGR1), calretinin (CR), and NeuN. Expression of the immediate-early gene EGR1 is increased by neuronal activation and is thus a proxy marker of neuronal activity [69]. Immature postmitotic neurons transiently express CR before these cells fully mature and begin to express the mature granule cell marker calbindin [70]. Colocalization of CR with NeuN, a neuronal marker [71], identifies immature granule neurons in the granule cell layer of the DG. The total number of active EGR1 + cells in the DG increased significantly in ketamine-treated mice (Fig. 2b, c), as did the number of NeuN + CR + cells (Fig. 2d). However, saline- and ketamine-treated mice had the same percentage of CR + cells that were activated, measured by EGR1 positivity (Fig. 2e). Conversely, the percentage of EGR1 + activated neurons that were CR + also was unaffected by multiple doses of ketamine (Fig. 2f). While the numbers of CR + EGR1- and CR-EGR1 + cells/mm^3^ increased after ketamine treatment, the number of activated immature neurons, CR + EGR1 + cells/mm^3^, was not significantly different (Supp. Figure 2). Taken together, these findings indicate that the increased activity in the DG at 14 DPI was not selective for the immature neuronal population (NeuN + CR +) that is involved in ketamine’s rapid effects [26].

### Multiple doses of ketamine increase AHN

**Fig. 3:**
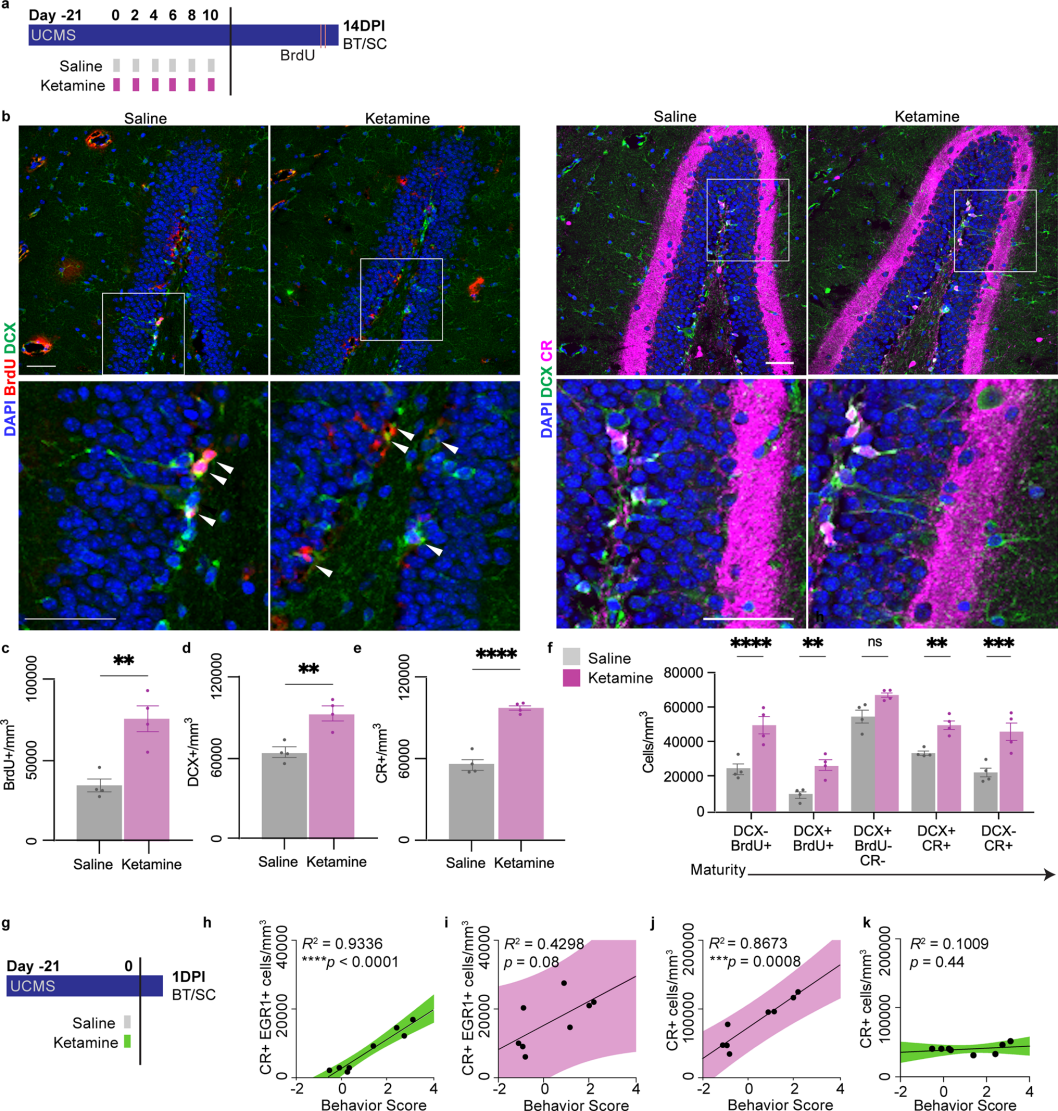
Multiple doses of ketamine increase adult hippocampal neurogenesis. **a** 8–10-week-old mice underwent unpredictable chronic mild stress (UCMS) and received 6 doses of subanesthetic (3 mg/kg) ketamine (K; purple) or saline (S; grey) by intraperitoneal injection prior to BT and SC for immunohistochemistry 14 days after the final injection (14DPI). BrdU was administered twice: 22 and 24 h prior to euthanasia (pink lines). **b** Representative IHC staining and colocalization of DAPI (blue, nuclear stain), BrdU (red), DCX (green), and CR (magenta) in the DG. Scale bars represent 50um. Arrows indicate BrdU + DCX + double-positive cells. White squares indicate the inset region shown in the second row. **c** BrdU + cells/mm^3^ were higher in ketamine-treated mouse DGs than in in saline-treated mouse DGs (*n* = 4 mice/group). Unpaired, two-tailed *t* test: **p = 0.0036. **d** DCX + cells/mm^3^ were higher in ketamine-treated mouse DGs than in in saline-treated mouse DGs (*n* = 4 mice/group). Unpaired, two-tailed *t* test: **p = 0.0039. **e** CR + cells/mm^3^ were higher in ketamine-treated mouse DGs than in in saline-treated mouse DGs (*n* = 4 mice/group). Unpaired, two-tailed *t* test: ****p < 0.0001. **f** Cells/mm^3^ in the immature neuronal lineage, marked by single- or double-labeling with BrdU, DCX, and/or CR, were higher in ketamine-treated mouse DGs than in in saline-treated mouse DGs (*n* = 4 mice/group). Markers are shown in order of association with cell stages, from less mature to more mature. Two-way Repeated Measures ANOVA: Maturation stage*Treatment ns *p* = 0.3018; Maturation stage ***p = 0.0001; Treatment ****p < 0.0001; Subject (individual mouse) ns *p* = 0.4321. Šídák's multiple comparisons test, S v K: BrdU + DCX- ****p < 0.0001, S v K: DCX + BrdU + **p = 0.0065, S v K: DCX + BrdU- CR- ns *p* = 0.0674, S v K: DCX + CR + **p = 0.0090, S v K: CR + DCX- **p = 0.0001. **g** 8–10-week-old male and female mice underwent UCMS (dark blue bar) and were given a single dose of subanesthetic (3 mg/kg) ketamine (K; green) or saline (S; grey) by intraperitoneal injection mice prior to BT and SC at 1DPI. **h** There was a highly significant correlation between behavior score and CR + EGR1 + cells/mm^3^ in mice given a single dose of saline or ketamine 1 day prior to BT and SC. Pearson *R* = 0.9663, ****p < 0.0001. The 95% Confidence Interval (CI) is shown in green. **i** There was no significant correlation between behavior score and CR + EGR1 + cells/mm^3^ in mice given multiple doses of saline or ketamine. Pearson *R* = 0.6556 ns *p* = 0.0776. The 95% CI is shown in purple. **j** There was a highly significant correlation between behavior score and CR + cells/mm^3^ in mice given multiple doses of saline or ketamine. Pearson *R* = 0.9313 ***p = 0.0008. The 95% CI is shown in purple. **k** There was no significant correlation between behavior score and CR + cells/mm^3^ in mice given a single dose of saline or ketamine 1 day prior to BT and SC. Pearson *R* = 0.3176 ns *p* = 0.4433 The 95% CI is shown in green

The increase in the number of DG immature neurons (NeuN + CR +) after multiple doses of ketamine (Fig. 2d) suggested an increase in AHN. To assess the effects of repeated ketamine injections on AHN more fully, BrdU was administered prior to BT and SC for IHC analysis of the number, cell cycle state, and progression of cells in the DG immature neuron lineage (Fig. 3a, b). Repeated ketamine administration more than doubled the number of BrdU + cells/mm^3^ (Fig. 3c) and significantly increased the numbers of both doublecortin + (DCX) neuronal progenitors (Fig. 3d) and CR + immature neurons (Fig. 3e). Co-labeling of these markers was performed to assess the relative maturity of cell populations in the DG; ketamine increased the numbers of BrdU + progenitors (DCX–, BrdU +), dividing neuroblasts (BrdU + DCX +), post-mitotic neuroblasts (DCX+/BRDU–/CR–), and late-immature neurons (DCX–/CR +) (Fig. 3f). The total number of cells in the granule neuron lineage, defined as BrdU, DCX, and/or CR positivity increased with ketamine treatment (Supp. Figure 3a). When scaled by the total number of cells in the granule neuron lineage/mm^3^ and normalized against saline, DCX–/BrdU + , DCX + /BrdU + , and DCX–/CR + populations increased, while DCX + /BRDU–/CR– and DCX + /CR + populations decreased (Supp. Figure 3b). Thus, repeated ketamine increased AHN both by increasing cell division of neuronal precursors as well as by accelerating maturation of the post-mitotic (BrdU–) DCX + pool to generate CR + immature neurons. By contrast, 24 h after a single dose of ketamine there was no significant change in the number of neuronal precursors (NeuroD1 + , or ND +) or immature neurons (CR +) (Supp. Figure 3c), and we previously showed that there is no change in the number of DCX + cells after a single dose of ketamine [26]. We thus conclude that while AHN is not altered by a single dose of ketamine [26], multiple doses of ketamine significantly increase AHN.

The rapid effects of ketamine on behavior require immature neuron activity [26]. We observed that 24 h after a single dose of ketamine (Fig. 3g), there is a strong correlation between the number of active immature neurons (CR + EGR1 + /mm^3^) and the behavior score (Fig. 3h). By contrast, 14 days after cessation of multiple ketamine doses, there is no significant correlation between the number of CR + EGR1 + neurons and ketamine’s sustained behavioral effect (Fig. 3i). Instead, the sustained behavioral effect after multiple ketamine doses is strongly correlated with the numbers of both CR + (Fig. 3j) and DCX + cells (Supp. Figure 3d), while there is no similar correlation for ketamine’s rapid effect (Fig. 3k). No correlation was observed between sustained behavioral effects and other groups of markers (Supp. Figure 3e), further reinforcing that it is not the activity, but rather the number of immature neurons that is relevant to the sustained behavioral effects of ketamine.

### Ketamine decreases BMP signaling in correlation with behavior

A single dose of ketamine increases the proportion of immature (CR +) neurons that express EGR1 without altering neuron numbers. By contrast, repeat doses increase the total number of immature neurons, and accordingly, the total number of active immature neurons, without changing the proportion of CR + cells that express EGR1. These findings suggest that the sustained behavioral effects of ketamine are mediated by a different mechanism than the rapid effects.

**Fig. 4:**
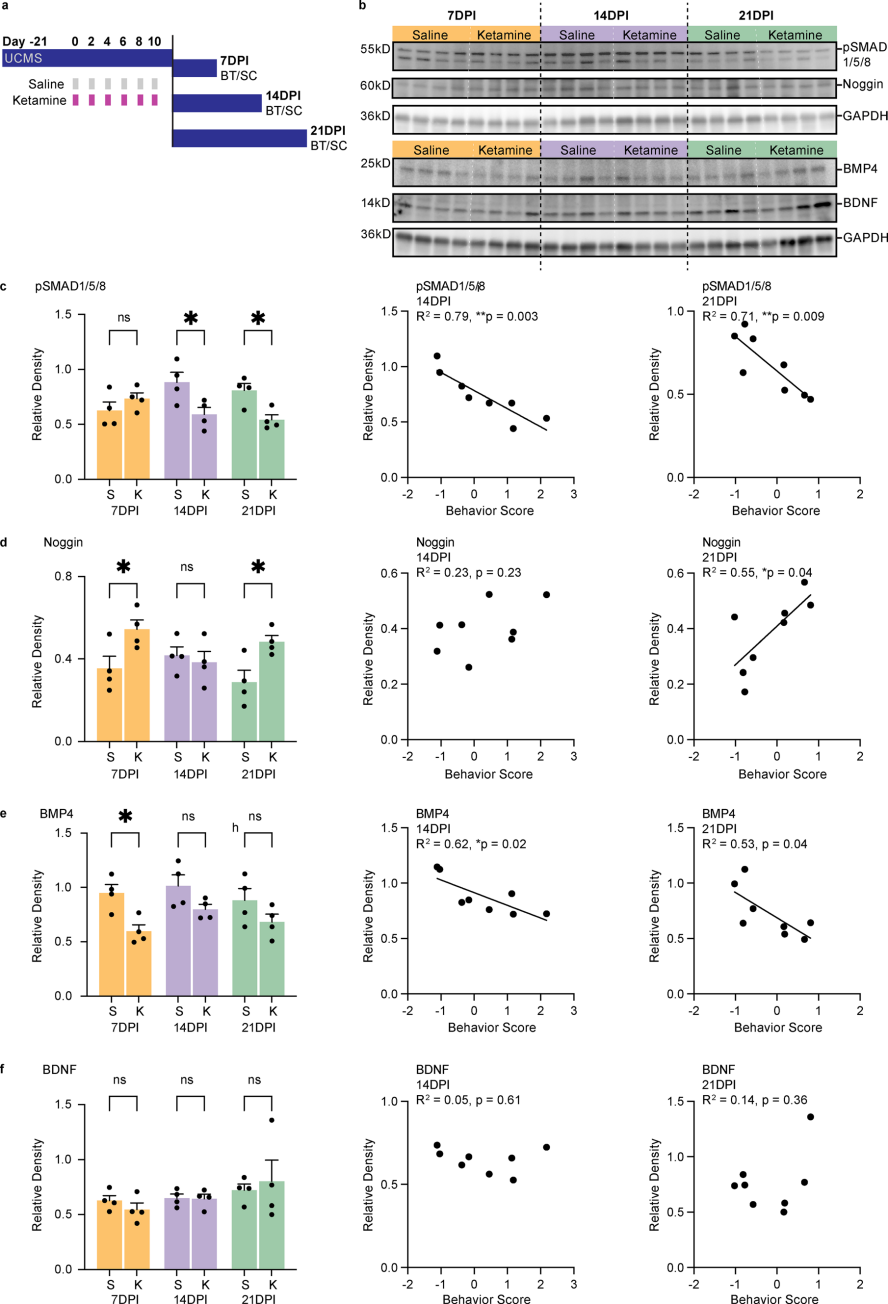
BMP signaling decreases after multiple doses of ketamine. **a** Timeline of UCMS and administration of multiple doses of subanesthetic (3 mg/kg) ketamine (purple) or saline (grey) by intraperitoneal injection in 8–10-week-old mice prior to BT and SC for western blots 7, 14, or 21 days after the final injection. **b** Representative western blots of dentate gyrus tissue probed for phospho-Smad1/5/8 (pSMAD1/5/8), Noggin, BMP4, BDNF and the loading control GAPDH at 7DPI (wells 1–8; yellow), 14DPI (wells 9–16; purple), and 21DPI (wells 17–24; green). Vertical dashed lines indicate different time points (black dashed line) or different treatment groups (white dashed line). Molecular weights are indicated in kilodaltons (kD). Blots were quantified by densitometric analysis. **c** The relative density of pSMAD1/5/8 versus the loading control GAPDH is significantly lower in ketamine-treated mice than in saline-treated mice at 14 and 21 DPI. Two-way ANOVA: Interaction *p = 0.0130, DPI ns *p* = 0.5999, ketamine *p = 0.0137; Šídák's multiple comparisons test, S (*n* = 4) v K (*n* = 4): 7DPI ns *p* = 0.6050, S (*n* = 4) v K (*n* = 4): 14DPI *p = 0.0194, S (*n* = 4) v K (*n* = 4): 21DPI *p = 0.0348. There was a significant correlation between behavior score and the relative density of pSMAD1/5/8 in the DG of mice given multiple doses of saline or ketamine ending 14 days prior to BT and SC. Pearson *R* = – 0.8905 **p = 0.003. There was a significant correlation between behavior score and the relative density of pSMAD1/5/8 in the DG of mice given multiple doses of saline or ketamine ending 21 days prior to BT and SC. Pearson *R* = – 0.8415 **p = 0.0088. **d** The relative density of Noggin is significantly higher in ketamine-treated mice than in saline-treated mice at 7 and 21DPI. Two-way ANOVA: Interaction ns *p* = 0.0508, DPI ns *p* = 0.4155, ketamine **p = 0.0094; Šídák's multiple comparisons test, S (*n* = 4) v K (*n* = 4): 7DPI *p = 0.0411, S (*n* = 4) v K (*n* = 4): 14DPI ns *p* = 0.9488, S (*n* = 4) v K (*n* = 4): 21DPI *p = 0.0350. There was no significant correlation between behavior score and the relative density of Noggin in the DG of mice given multiple doses of saline or ketamine ending 14 days prior to BT and SC. Pearson *R* = – 0.4833 ns *p* = 0.2251. There was a significant correlation between behavior score and the relative density of Noggin in the DG of mice given multiple doses of saline or ketamine ending 21 days prior to BT and SC. Pearson *R* = 0.7392 *p = 0.0361. **e** The relative density of BMP4 is significantly lower in ketamine-treated mice than in saline-treated mice at 7DPI.Two-way ANOVA: Interaction ns *p* = 0.05841, DPI ns *p* = 0.2096, ketamine **p = 0.0011; Šídák's multiple comparisons test, S (*n* = 4) v K (*n* = 4): 7DPI *p = 0.0186, S (*n* = 4) v K (*n* = 4): 14DPI ns *p* = 0.2110, S (*n* = 4) v K (*n* = 4): 21DPI ns *p* = 0.2685. There was a significant correlation between behavior score and the relative density of BMP4 in the DG of mice given multiple doses of saline or ketamine ending 14 days prior to BT and SC. Pearson *R* = – 0.7893 *p = 0.0199. There was a significant correlation between behavior score and the relative density of BMP4 in the DG of mice given multiple doses of saline or ketamine ending 21 days prior to BT and SC. Pearson *R* = – 0.7269 *p = 0.0411. **f** The relative density of BDNF is not significantly different in ketamine-treated mice than in saline-treated mice at 7, 14, or 21DPI. Two-way ANOVA: Interaction ns *p* = 0.05841, DPI ns *p* = 0.2096, ketamine **p = 0.0011; Šídák's multiple comparisons test, S (*n* = 4) v K (*n* = 4): 7DPI ns *p* = 0.8944, S (*n* = 4) v K (*n* = 4): 14DPI ns *p* > 0.9999, S (*n* = 4) v K (*n* = 4): 21DPI ns p = 0.9015. There was no significant correlation between behavior score and the relative density of BDNF in the DG of mice given multiple doses of saline or ketamine ending 14 days prior to BT and SC. Pearson *R* = – 0.2141 ns *p* = 0.6107. There was no significant correlation between behavior score and the relative density of BDNF in the DG of mice given multiple doses of saline or ketamine ending 21 days prior to BT and SC. Pearson *R* = 0.3744 ns *p* = 0.3609. Western blot data are presented as means ± s.e.m. and analyzed by two-tailed Student’s *t* test. Values were normalized to GAPDH. Correlation data are presented as linear regressions and analyzed by Pearson R correlation coefficients and two-tailed *p* values. Linear regression lines are only shown for *p* values < 0.05

Conventional antidepressants require an increase in neuron number through AHN to exert sustained behavioral effects [35, 41–43], and inhibition of BMP signaling is a common final pathway that mediates the effects of multiple classes of antidepressants on both AHN and behavior [45]. We, therefore, hypothesized that a similar mechanism might underlie the sustained effects of multiple ketamine doses. BMP signaling involves binding of a BMP ligand to membrane receptors that phosphorylate SMAD1/5/8 (pSMAD1/5/8), which is then translocated to the nucleus where it regulates the transcription of BMP target genes, including genes that reduce the proliferation of neural stem/progenitor cells [72]. We, therefore, measured levels of pSMAD1/5/8 in the microdissected DG after repeated doses of ketamine and examined the correlation with behavior at each time point (7, 14, and 21 DPI; Fig. 4a–c). At 14 and 21DPI, pSMAD1/5/8 levels in ketamine-treated mouse DGs were significantly decreased relative to saline-treated mouse DGs (Fig. 4b, c). At these time points, levels of pSMAD1/5/8 were also tightly negatively correlated with behavior scores. We then probed for additional proteins involved in BMP signaling: BMP4, a common BMP ligand in the dentate gyrus, and Noggin, a competitive antagonist of BMP4 [45–47, 72–74]. We also probed for BDNF, a molecule associated with hippocampus-dependent antidepressant effects [23–25, 75, 76]. Noggin was significantly increased at 7 and 21DPI in the ketamine groups (Fig. 4d). BMP4 was decreased in the ketamine group at 7DPI (Fig. 4e). There were no significant differences in BDNF levels between saline and ketamine groups at 7, 14, or 21DPI (Fig. 4f). At both 14 and 21DPI, proteins involved with BMP signaling were tightly correlated with behavior; BMP4 and pSMAD1/5/8 negatively correlated with behavior, while Noggin was associated with higher behavior scores (Fig. 4c–e). At these subacute time points, BDNF was not significantly correlated with behavior scores (Fig. 4f). We also performed immunohistochemistry to examine the distribution and intensity of expression between groups at 14DPI (Supp. Figure 4).

### Increasing BMP levels decreases AHN and blocks ketamine’s sustained effects without affecting ketamine’s rapid effects

**Fig. 5:**
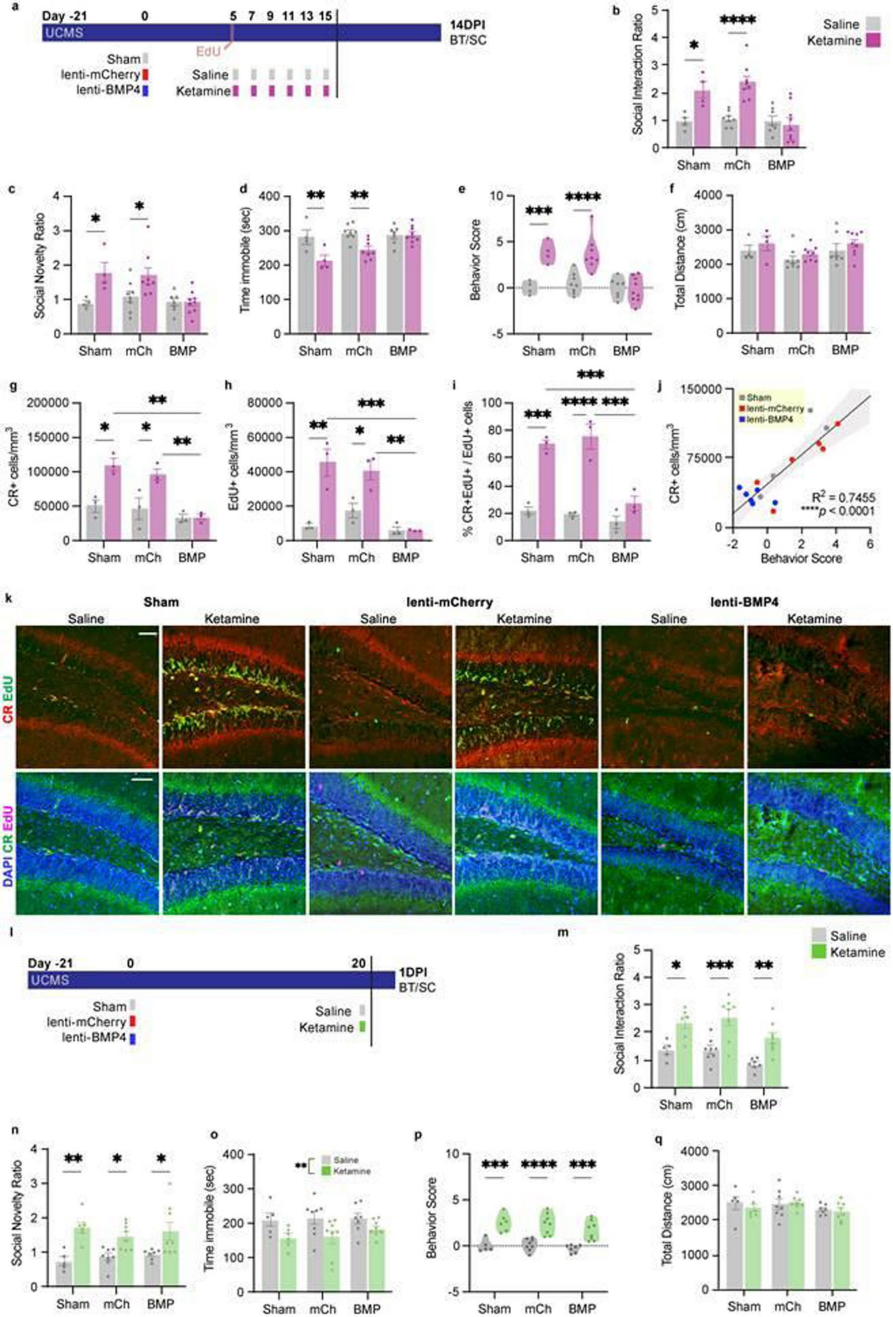
Increased BMP levels prevent sustained effects of ketamine on neurogenesis and behavior. **a** Experimental timeline for lentiviral injections and repeated ketamine dosing. Control virus (lenti-mCherry) or BMP4 overexpression virus (lenti-BMP4) were injected after 21 days of UCMS. Day 5 after viral injections, six doses of saline (grey) or ketamine (purple) were administered from Day 5-Day 15. BT and SC occurred 14 days after the final injection (14DPI). **b** BMP4 overexpression blocked ketamine’s effect on the Social Interaction Ratio. Two-way ANOVA: Interaction *p* = 0.0015, Virus *p* = 0.0006, Ketamine *p* = 0.0001. Šídák's multiple comparisons test: Sham S (*n* = 4) v K (*n* = 4) *p = 0.0165, mCh S (*n* = 8) v K (*n* = 8) ****p < 0.0001, BMP S (*n* = 7) v K (*n* = 9) ns *p* = 0.9671. **c** BMP4 overexpression blocked ketamine’s effect on the Social Novelty Ratio. Two-way ANOVA: Interaction *p* = 0.0537, Virus *p* = 0.0104, Ketamine *p* = 0.0019. Šídák's multiple comparisons test: Sham S (*n* = 4) v K (*n* = 4) *p = 0.0241, mCh S (*n* = 8) v K (*n* = 8) *p < 0.0276, BMP S (*n* = 7) v K (*n* = 9) ns *p* > 0.9999. **d** BMP4 overexpression blocked ketamine’s effect on Time immobile (sec) in the TST. Two-way ANOVA: Interaction **p = 0.0096, Virus **p = 0.0076, Ketamine ***p = 0.0002. Šídák's multiple comparisons test: Sham S (*n* = 4) v K (*n* = 4) **p = 0.0046, mCh S (*n* = 8) v K (*n* = 8) **p = 0.0028, BMP S (*n* = 7) v K (*n* = 9) ns *p* = 0.9996. **e** There is no effect of ketamine on the behavior scores of BMP4-treated mice. Two-way ANOVA: Interaction ***p = 0.0008, Virus ***p = 0.0001, Ketamine ****p < 0.0001. Šídák's multiple comparisons test: Sham S (*n* = 4) v K (*n* = 4) ***p = 0.0009, mCh S (*n* = 8) v K (*n* = 8) ****p < 0.0001, BMP S (*n* = 7) v K (*n* = 9) ns *p* = 0.9882. **f** There were no locomotor differences as assessed by the total distance (cm) travelled in the OFT. Two-way ANOVA: Interaction *p* = 0.9812, Virus *p* = 0.0710, Ketamine *p* = 0.1049. Šídák's multiple comparisons test: Sham S (*n* = 4) v K (*n* = 4) *p* = 0.7454, mCh S (*n* = 8) v K (*n* = 8) *p* = 0.7186, BMP S (*n* = 7) v K (*n* = 9) ns *p* = 0.6410. **g** BMP4 overexpression blocked the ketamine-induced increase in CR + cells/mm^3^. Two-way ANOVA: Interaction *p* = 0.0199, Virus ***p = 0.0007, Ketamine ***p = 0.0005. Šídák's multiple comparisons test: *n* = 3/group Sham S v K *p = 0.0114, Sham K v BMP K **p = 0.0014, mCh S v mCh K *p = 0.0439, mCh K v BMPK **p = 0.0085. **h** BMP4 overexpression blocked the ketamine-induced increase in EdU + cells/mm^3^.Two-way ANOVA: Interaction **p = 0.0045, Virus ***p = 0.0004 Ketamine ***p = 0.0002. Šídák's multiple comparisons test: *n* = 3/group Sham S v K **p = 0.0013, Sham K v BMP K ***p = 0.0007, MCh S v MCh K *p = 0.0492, mCh K v BMPK **p = 0.0021. **i** BMP4 overexpression reduced the percentage of EdU + cells that were also CR + . Two-way ANOVA: Interaction **p = 0.0024, ***p = 0.0002, Ketamine ****p < 0.0001. Šídák's multiple comparisons test: *n* = 3/group Sham S v K ***p = 0.0002, Sham K v BMP K ****p = 0.0007, MCh S v MCh K ****p < 0.0001, mCh K v BMPK ***p = 0.0002. **j** There was a significant correlation between behavior score and CR + cells/mm^3^. Grey dots indicate sham-group mice, red dots indicate lenti-mCh-treated mice, and blue dots indicate lenti-BMP4-treated mice. ****p < 0.0001. **k** Representative IHC staining and colocalization of EdU (top row, green; bottom row, magenta), CR (top row, red; bottom row, green), and DAPI (bottom row, blue) in the DGs of saline and ketamine-treated mice in the lenti-mCh, lenti-BMP, and sham groups. Scale bars represent 50um. **l** Experimental timeline for lentiviral injections with a single dose of ketamine. Viral injections were conducted as before. Mice received one dose of saline (grey) or ketamine (green) and underwent BT and SC at 1DPI. **m** BMP overexpression did not block effect of a single dose of ketamine on the Social Interaction Ratio at 1DPI. Two-way ANOVA: Interaction *p* = 0.8058, Virus **p = 0.0049, Ketamine *p* < 0.0001. Šídák's multiple comparisons test: Sham S (*n* = 5) v K (*n* = 6) *p = 0.0119, mCh S (*n* = 8) v K (*n* = 8) *p = 0.0002, BMP S (*n* = 7) v K (*n* = 8) **p = 0.0039. **n** BMP overexpression did not block the effect of a single dose of ketamine on the Social Novelty Ratio at 1DPI. Two-way ANOVA: Interaction *p* = 0.4976, Virus *p* = 0.7494, Ketamine *p* < 0.0001. Šídák's multiple comparisons test: Sham S (*n* = 5) v K (*n* = 6) *p = 0.0016, mCh S (*n* = 8) v K (*n* = 8) *p = 0.0253, BMP S (*n* = 7) v K (*n* = 8) *p = 0.0127. **o** There was a significant ketamine effect on Time immobile (sec) in the TST at 1DPI. Two-way ANOVA: Interaction ns *p* = 0.6707, Virus ns *p* = 0.6355, Ketamine **p = 0.0022. Šídák's multiple comparisons test: Sham S (*n* = 5) v K (*n* = 6) ns *p* = 0.1499, mCh S (*n* = 8) v K (*n* = 8) ns *p* = 0.0531, BMP S (*n* = 7) v K (*n* = 8) ns *p* = 0.5216. **p** There is no effect of the BMP lentivirus on the behavior scores of mice given one dose of ketamine 24 h prior to BT. Two-way ANOVA: Interaction *p* = 0.7073, Virus *p* = 0.1487, Ketamine ****p < 0.0001. Šídák's multiple comparisons test: Sham S (*n* = 5) v K (*n* = 6) ***p = 0.0001, mCh S (*n* = 8) v K (*n* = 8) ****p < 0.0001, BMP S (*n* = 7) v K (*n* = 8) ns ***p = 0.0004. **q** Total distance (cm) travelled in the OFT. Two-way ANOVA: Interaction *p* = 0.7151, Virus *p* = 0.1896, Ketamine *p* = 0.5873. Šídák's multiple comparisons test: Sham S (*n* = 5) v K (*n* = 6) *p = 0.8022, mCh S (*n* = 8) v K (*n* = 8) *p = 0.9880, BMP S (*n* = 7) v K (*n* = 8) ns *p* = 0.9848

Due to the significant correlations between proteins involved in the BMP signaling pathway and behavior, we asked whether decreased BMP signaling may mediate the sustained behavioral effects of multiple doses of ketamine, similar to mechanisms that mediate the effects of conventional antidepressants [45, 47]. We, therefore, tested whether preventing the reduction in BMP signaling after repeat ketamine doses blocks effects of the drug on AHN and on behavior (Fig. 5a). Mice were exposed to UCMS, and the DG was then stereotaxically injected either with a lentiviral construct including BMP4 and a mCherry fluorescent tag (hereafter called “lenti-BMP4”) [47] or with a control vector containing only the mCherry tag (“lenti-mCherry” or “mCh”). Another group received sham surgery with no viral injection. Multiple doses of ketamine or saline injections began 5 days post lentiviral injection, and mice were also given two EdU injections on day 5 to label proliferating cells and their progeny. Two weeks after the last dose of saline or ketamine, the mice underwent BT and were euthanized for sample collection.

In both the sham and lenti-mCherry control groups, repeat ketamine treatment-induced improvements in performance on the SIT, SNT, and TST (Fig. 5b–d) as well as the overall behavior score (Fig. 5e). However, the effects of ketamine on behavior were blocked in the lenti-BMP4 (BMP) group. There were no significant changes in overall locomotor activity in any of the groups (Fig. 5f).

In parallel with preventing ketamine’s sustained behavioral effects, the lenti-BMP4 injection also blocked changes in AHN (Fig. 5g–k). IHC examination for mCherry showed that there was a widespread expression of the viral vector throughout the DG in both the lenti-BMP4 and lenti-mCherry groups (Supp. Figure 5). In both the sham and lenti-mCherry groups, mice that received repeated doses of ketamine had significantly increased numbers of CR + cells (Fig. 5g) and EdU + cells (Fig. 5h), and more of the EdU + cells were also CR + (Fig. 5i). However, the effects of ketamine on AHN were blocked in the lenti-BMP4 group. As before, the number of CR + cells/mm^3^ was tightly correlated with behavioral scores (Fig. 5j). Thus, prevention of the decrease in BMP signaling blocked the effects of repeated doses of ketamine on both AHN and behavior.

To examine whether BMP signaling is involved in the rapid behavioral effects of a single dose of ketamine, the DG was injected with vectors as in the prior experiment, and on day 20, mice were given a single dose of saline or ketamine 24 h prior to BT (Fig. 5l). Ketamine treatment produced similar antidepressant-like behavioral effects in all 3 groups, with no change upon BMP overexpression (Fig. 5m–q). Thus, while decreased BMP signaling with an attendant increase in AHN mediates the more sustained effects of repeat ketamine dosing, it is not involved in the rapid onset effects of the drug.

## Discussion

A single dose of ketamine exerts rapid antidepressant effects, but the effects wane rapidly. Repeated dosing maintains the antidepressant effects and extends the duration of effects after cessation of treatment. However, repeated administration may be addictive and is associated with significant medical risks [10–12]. There is therefore a great need to discover new molecular targets for antidepressant action and to develop safer drugs that produce similar rapid as well as sustained antidepressant responses. Of particular interest would be the ability to rapidly induce and then maintain behavioral improvements alone or until conventional antidepressants with good safety profiles can take effect.

We previously observed that the rapid onset effects of ketamine are mediated by an increase in the activity of adult-born immature dentate granule neurons without any change in the number of these cells [27]. Here, we observed that multiple doses of ketamine significantly increase AHN, and that unlike ketamine’s rapid effects, the sustained effects do not correlate with a preferential increase in immature neuron activity. Instead, as with many conventional antidepressants [45], administration of multiple doses of ketamine decreased levels of pSMAD1/5/8, the main secondary messenger that mediates BMP signaling. Ketamine also increased levels of the BMP antagonist Noggin, at 7 and 21 days, while the effect was not significant at 14. While the decrease in the BMP4 ligand itself was observed only on Day 7, BMP4 is only one of the BMP ligands present in the hippocampus, and the levels of the ligand are not directly equitable to BMP signaling; the increase in Noggin and decrease in pSMAD1/5/8 suggests that BMP signaling as a whole is decreased in the niche.

Maintaining high levels of BMP signaling blocked the sustained, but not the rapid, behavioral effects of the drug. Thus, ketamine’s rapid and sustained behavioral effects occur through distinct mechanisms. Further, while the rapid onset effects of ketamine are not shared by conventional antidepressants, multiple doses of ketamine produce more sustained behavioral effects that share a common mechanism with other classes of antidepressants [45].

Fourteen days after the final ketamine dose, we observed an increase in multiple stages of AHN, including proliferative (BrdU +) stem and progenitor cells and post-mitotic immature DCX-/CR + neurons. The increase in DCX–/CR + cells occurs too rapidly for them to be derived from the early stem/progenitor cells, suggesting that the preexisting DCX pool is drawn upon to generate immature neurons concurrently with the increase in proliferative stem/progenitor cells. This is consistent with the observation of a relative reduction of DCX + /CR- cells (Supp. Figure 3b) after repeated injection and with prior observations that BMP signaling regulates the tempo of AHN [72]. One prior study found that a single dose of ketamine produced a short-lived increase in progenitor cell proliferation that was nevertheless unrelated to the behavioral effects of the drug [77]. One day after a single injection (1 DPI) we do not see any increase in immature neurons marked by NeuroD1 and Calretinin, which is consistent with the multi-week time course of AHN.

Inhibiting or blocking BMP signaling is sufficient to promote the reentry of quiescent cells into the cell cycle followed by differentiation of the cells [46, 72], which may explain the observed increases in both progenitor cells and immature neurons after ketamine treatment. However, neuroblasts experience significant selection and cell death as they reach the immature neuron stage and begin to integrate into the hippocampus [78]. The increased number of immature neurons that we observed thus also could reflect increased cell survival.

Immature neuron survival may be a mechanism by which BDNF also contributes to ketamine’s antidepressant effects. BDNF has been implicated in the rapid antidepressant effects of ketamine, but we did not observe a significant increase in BDNF with ketamine treatment at our time points. It has previously been reported that rodent BDNF expression rises within 24 h of ketamine treatment and normalizes within 24–72 hours [23, 76, 79, 80]. This is consistent with our findings showing no change in BDNF levels two weeks after the final ketamine treatment. We hypothesize that BDNF likely peaks rapidly, then normalizes, whereas downstream effects persist and contribute to a niche that is more permissive of increased immature neuron survival [23, 81]. Paired with increased neuroblast proliferation due to decreased BMP signaling, enhanced cell survival mediated by the downstream effects of BDNF signaling may synergize to support the neurogenic effects of multiple ketamine injections. Future cell lineage tracing studies could provide specific information on the effects of multiple ketamine doses on cell survival in the subgranular zone of the DG.

In our study, viral overexpression of BMP4 produced the expected reduction in proliferation of dividing cells in comparison to saline-treated groups, but it did not elicit increased stressed behavior. Although decreased BMP signaling is associated with improved affective behavior both in this study and others, there are conflicting reports on whether the converse also holds, i.e. if increasing BMP signaling worsens mouse stress behavior [45, 47]. There is variation in whether the studies were conducted in mice that were either stress-naïve or that underwent shorter UCMS paradigms. The UCMS paradigm itself increases BMP signaling [45], and it is possible that there is a plateau in the behavioral effects of further increases in BMP signaling. Alternatively, the behavioral tests themselves may not be sensitive enough to detect these changes.

How the change in the number of immature neurons induces alterations in stress behavior remains an open question. The number of immature neurons generated in response to ketamine or other antidepressants is relatively small compared to the overall pool of dentate granule neurons, but the effects on behavior are dramatic. Immature neurons are known to be more excitable, show higher neuroplasticity, and have different neurotransmitter profiles [82–87]. We hypothesize that along with the increase in the number of immature granule neurons resulting from sustained ketamine treatment, there may be alterations in immature neuron morphology and circuitry. In addition to the known role of BMP signaling in regulating proliferation, modulation of BMP signaling also alters structural and electrophysiological properties of immature neurons [72, 88, 89], thus potentially changing the circuitry of immature neurons both within the DG and with their pre- and post-synaptic connections beyond the DG. Stress exposure also alters the circuitry and morphology of immature neurons [90]. Future studies may examine whether the synaptic interactions of immature neurons born in response to ketamine resemble what is observed with other antidepressants, and in turn, whether these immature neurons regulate existing physiological circuits or suppress aberrant ones.

Our study describes a mechanism underlying the sustained effects of ketamine following multiple injections and contributes to a nuanced view that the sustained effects of ketamine likely involve different molecular mechanisms than those driving its initial rapid effects.

In a clinical setting, ketamine is often administered every other day for up to two weeks, leading to antidepressant effects that last multiple weeks after the final injection [7–9], but there is limited information on how this prolonged effect occurs. Without mechanistic research, there is no way to fully address open clinical questions such as the ideal dosing scheme of ketamine, whether to administer conventional antidepressants in combination with ketamine, or how different conventional treatments may synergize with or inhibit the therapeutic effects of ketamine. Understanding ketamine’s dual mechanisms may inform the development of new therapeutic approaches with safe, rapid, and sustained antidepressant effects.

## Methods

### Mouse lines

All animal procedures were approved by the Northwestern University Institutional Animal Care and Use Committee (IACUC). All experiments were performed in accordance with the Public Health Service Policy on Humane Care and Use of Laboratory Animals. All animals were housed 3–5 per cage on a 14:10 h light: dark cycle in a controlled environment and received food and water ad libitum. Mice were housed within the following limits: acceptable temperature range of 64–79 °F (typically kept at 72–74 °F); humidity range: 30–70%. All BT was conducted during the light period.

For Figs. 1, 2, 3, we used male and female mice in each group. The analyses were first conducted to compare behavioral effects by sex (Supp. Figure 1), and as there were no behavioral differences found between the sexes, we reported the results for both sexes together. We used 8- to 10-week-old C57Bl/6 male and female mice (Charles River, Wilmington, MA, USA), Mice were randomly assigned to experimental groups: saline or ketamine treatment, and single or multiple doses (Fig. 1). For Figs. 4 and 5, because we had observed no significant sex differences (Supp. Figure 1), we used female mice to avoid the fighting observed among chronically stressed males. At least three independent cohorts of mice were run for every experiment.

### Drug administration

Mice were weighed prior to drug administration. Ketamine (100 mg/ mL, Henry Schein NDC# 11,695-0702-1) was diluted in normal saline to a working concentration of 0.6 mg/mL and administered intraperitoneally (i.p.) at a 3 mg/kg dose. Ketamine and saline controls were administered at a volume of 100 uL per 20 g mouse. Mice received either a single injection of saline or ketamine or 6 injections over a 10-day period, i.e., one dose every other day.

For neuronal labeling in Figs. 1, 2, 3, Bromodeoxyuridine (BrdU, Sigma Aldrich, St. Louis, MO, USA) was dissolved in PBS. Mice received two i.p. injections of 50 mg/kg BrdU: one at 22 h and one at 24 h prior to euthanasia. For neuronal labeling in Fig. 5, EdU was dissolved in PBS. Mice received two i.p. injections of 25 mg/kg EdU, given two hours apart on day 5, and were sacrificed after completion of the experimental timeline.

### Viral vector production and stereotaxic viral injection

Lentiviral vectors were produced as previously described [45, 47, 72]. An internal ribosome entry site (IRES2) and the fluorescent protein mCherry were cloned into the pBOB lentiviral vector (addgene #12,337) to produce a pBOB-IRES2-mCherry control vector (Lenti-mCherry). A BMP4 overexpression vector, LV-secBMP4-IRES2-mCherry (Lenti-BMP4), was produced by inserting BMP4 cDNA with an N-terminal secretion tag into the pBOB-IRES2-mCherry backbone. The lentiviral particles were produced by the NU GetIN Viral Production Core.

Stereotaxic virus injections were performed using a Motorized Mouse Stereotaxic Instrument (Stoelting Co., Wood Dale, IL, USA), Quintessential Stereotaxic Injector (Stoelting Co., Wood Dale, IL, USA), and a microsyringe (Hamilton, 5 μL, NEUROS Model, 33ga., Blunt). Mice were anesthetized via inhalation of isoflurane. A midline scalp incision was made, and two holes were drilled into the skull. 2 μL of lentivirus was injected at a rate of 0.5 μl per minute into the bilateral dentate gyri at the following coordinates relative to bregma: 2 mm posterior, 1.5 mm lateral, 2 mm ventral. Five days after the viral injections, mice were treated with EdU and either a single dose of saline or ketamine, or multiple doses of ketamine or saline over two weeks. After BT and SC, lentiviral infection was confirmed by immunohistochemistry, and mice lacking successful virus infection in the bilateral DG were excluded from the analysis.

### Unpredictable chronic mild stress (UCMS)

All mice were subjected to unpredictable chronic mild stress (UCMS) for 3 weeks before each experiment and for the duration of each experiment, except for days when mice received IP injections or during the 5-day recovery period after the stereotaxic viral injection, as both of those procedures can be innately stressful.

Before exposure tostressors, animals were transferred to a clean room used for UCMS. Mice underwent exposure to 1 or 2 common stressors every day for a minimum of 2 h, except light cycle disturbances and restraint stress [44]. Stressors were administered on a randomized schedule, as before [26, 44]. After each daily stress period, animals were moved to clean cages and returned to the housing facility.

### Behavioral testing

For all behavioral analyses, mice were transferred to the testing room 1 h prior to testing to facilitate acclimation to the test environment. Every behavioral apparatus was wiped with 70% ethanol prior to each trial. The Social Interaction Test (SIT) and Social Novelty Test (SNT) were performed at the Northwestern University Behavioral Phenotyping Core Facility. The Tail Suspension Test (TST) was performed at the satellite area of the Kessler lab. All behavioral tests were performed by a female experimenter blinded to the experimental condition. Animals were sacrificed immediately after their last behavioral test to collect brain samples and tissue.

#### Three-chamber apparatus and sociability tests

Preference for social interaction and social novelty were evaluated using a three-chamber sociability test, where a center chamber separates two identical side chambers. First, the test mouse was habituated to the 3-chamber apparatus for 5 min. Distance traveled during the habituation period was recorded. For the Social Interaction Test (SIT), a wired cup containing an unfamiliar mouse and another cup with a small mouse-like object were introduced into the two identical side chambers. The test animal was allowed to explore the 3 chambers freely for 10 min. At the end of the exploration period, the test mouse was guided to the center chamber while the apparatus was cleaned, and the wired cups in the side chambers were replaced. The Social Novelty Test (SNT) occurred immediately after, this time with a new unfamiliar mouse in one cup instead of the object, and the original unfamiliar mouse in the other cup. The test animal was again allowed to explore the 3 chambers freely for 10 min. At the end of these 10 min, each mouse (test and unfamiliar) was returned to its respective home cage. In each set of experiments, the orientation of the two wired cups containing the unfamiliar mouse or the object was counterbalanced. The familiar mouse cup during the SNT was always placed on the opposite side as for the SIT. The movement of the test mouse and its time spent interacting with each wired cup were tracked and recorded using LimeLight 4 software (Actimetrics, Coulbourn Instruments) under white light at an intensity of 250 lx. Video recordings of the tests were quantified by an experimenter blind to the experimental condition.

The Social Interaction Ratio was calculated as the amount of time spent interacting with the unfamiliar mouse divided by the time spent interacting with the object. The Social Novelty Ratio was calculated as the amount of time spent interacting with the unfamiliar mouse divided by the time spent interacting with the familiar mouse.

All experimental mice that were housed in the same cage underwent testing at the same time to avoid interactions between tested and untested mice. All unfamiliar mice were age- and sex- matched with test mice, and were weight-matched within ± 1 g of each test mouse. Unfamiliar mice were from different litters than the test mice and had never interacted with them prior to the experimental test.

#### Tail suspension test (TST)

Tape was affixed to the test mouse's tail 2 cm from the tip and the mouse was suspended from a horizontal bar at a height of 30 cm. Testing was conducted under white light at an intensity of 250 lx. The mouse was suspended for 6 min. Video recordings of the test were quantified by an observer blind to the experimental condition. The total time spent in an immobile posture by each mouse was measured.

#### Open field test (OFT)

The OFT apparatus consisted of a 56 × 56 cm open arena with 30 cm high walls under white light (250 lx). The mouse was placed into the center of the arena and allowed to move freely for 5 min. Activity was recorded and tracked by LimeLight 3 software (Actimetrics, Coulbourn Instruments). The software recorded and analyzed the total distance traveled in the arena.

### Behavior score

Z-scores allow for the standardization of multiple behaviors into a combined behavioral score as described previously [60, 61]. Z-score values were calculated for each mouse for the social interaction ratio (SIT), social novelty ratio (SNT), and time immobile (TST). The saline-treated 1 DPI group was the control group for the single-dose regimen. The saline-treated 7 DPI group was the control group for the multiple-dose regimen. The directionality of scores in the TST were adjusted so that positive score values reflected less stress behavior. Individual scores for each test were then averaged per the formula below for each individual mouse, to balance potential biases associated with each test.

$${\text{Behavior score }} = \, \left[ {z_{{{\text{SIT}}}} + \, z_{{{\text{SNT}}}} + \, \left( { - z_{{{\text{TST}}}} } \right)} \right]/ \, \left( {{\text{number of tests}},{\text{ i}}{\text{.e}}{.}, \, 3} \right)$$

### Perfusion

Mice were transcardially perfused with PBS. Brains were hemisected at the midline; one half was fixed for 24 h in 4% paraformaldehyde for immunohistochemistry, and the DG was microdissected from the other half for future immunoblot analysis.

### Protein extraction and western blotting

The DG was micro-dissected from PBS-perfused half-brains, and the tissue was mechanically homogenized on ice in T-per protein extraction reagent (Thermo Fisher Scientific, Waltham, MA, USA) with Halt protease and phosphatase inhibitors (Thermo Fisher Scientific). Lysates were centrifuged at 10,000 rpm for 15 min at 4 °C and the supernatant was collected for western blot analysis.

Western blotting was performed using standard techniques. Protein was quantified via BCA protein assay (Thermo Fisher Scientific, Waltham, MA, USA) and samples were heated at 100 °C for 5 min in denaturing buffer. 20–50 μg of protein was loaded onto a 4%-20% polyacrylamide gel. Following electrophoresis, protein was transferred to a polyvinylidene fluoride membrane (Millipore, Burlington, MA, USA) for one hour at 4 °C. The membrane was blocked in 5% bovine serum albumin (BSA) (Sigma Aldrich, St. Louis, MO, USA) in TBS with 0.1% Tween-20 (TBS-T) for 1–2 h at room temperature (RT) and incubated at 4 °C overnight in primary antibody diluted in blocking solution. The membrane was then washed three times in TBS-T and incubated for 1 h at RT with a horseradish peroxidase-conjugated secondary antibody (Santa Cruz Biotechnology, Dallas, TX, USA) diluted 1:2000 in blocking buffer. Following three final washes in TBS-T, chemiluminescence was detected using Radiance Western Blotting substrates (Thermo Fisher Scientific, Waltham, MA, USA) and the Azure Biosystems C600 imaging system.

Primary antibodies were rabbit anti-phospho-Smad1/5/8 (1:500, AB3848, Millipore), mouse anti-BMP4, (1:1000, AM01363 PU-N, OriGene Technologies), mouse anti-noggin (1:1000, TA500116, OriGene Technologies), rabbit anti-BDNF (1:1000, #47,808, Cell Signaling), rabbit anti-total Smad (1:1000, NB-100-56656, Novus Biologicals), mouse anti-GAPDH (1:2000, MCA-1D4, Encor),and mouse anti-GAPDH (1:4000, MAB374, Millipore).

### Immunohistochemistry (IHC)

Perfused half-brains were fixed overnight in 4% paraformaldehyde and then transferred to 30% sucrose for 24 h. Forty-micrometer thick floating sections were obtained using a microtome (Microm HM 450, Thermo Fisher, Waltham, MA, USA). Tissue sections were washed three times in PBS, blocked in 10% normal serum with 0.25% Triton X-100 in PBS for 1 h at room temperature (RT), and incubated overnight at 4 °C in the primary antibody diluted in 1% bovine serum albumin (BSA) with 0.25% Triton X-100 in PBS. Sections were washed three times in PBS and then incubated for 1 h at RT with a fluorophore-conjugated secondary antibody (Alexa-488, Alexa-555, or Alexa-647, ThermoFisher Scientific) and 4′,6-diamidino-2-phenylindole (DAPI) for nuclear stain (Invitrogen Hoechst 33,258, Carlsbad, CA, USA). Following three final washes in PBS, floating sections were mounted with ProLong Gold Antifade Reagent (Life Technologies, Carlsbad, CA, USA).

For BrdU detection, floating sections were incubated in 10 mM sodium citrate buffer with 0.05% Tween-20, pH 6.0 for 20 min at 95 °C, cooled to RT, washed three times in PBS, and transferred to 2 N HCl for 30 min at RT. Sections were then quenched in 0.1 M sodium tetraborate, pH 8.5 for 10 min at RT and washed in PBS. Blocking and antibody incubation were then performed as described above.

Detection of the EdU cell proliferation marker was performed according to the kit instructions (Click-iT EdU Cell Proliferation Kit for Imaging with Alexa Fluor 647 dye: C10349, ThermoFisher Scientific). Sections underwent primary and secondary staining as above, were washed three times in PBS after incubation in secondary antibodies and were then incubated with the Click-it EDU Alexa Fluor cocktail for 1 h at RT, while protected from light. Following three final washes in PBS, floating sections were mounted with ProLong Gold Antifade Reagent (Life Technologies, Carlsbad, CA, USA).

#### Primary antibodies

Chicken anti-Calbindin (1:1000, #CPCA, EnCor), guinea pig anti-Doublecortin (1:500, AB2253, Millipore), rabbit anti-EGR1 (1:500, #4153, Cell Signaling), mouse anti-NeuN (1:500, MAB377, Millipore), guinea pig anti-Calretinin (1:1000, CRgp7, Swant), rat anti-BrdU (1:250, ab6326, Abcam), and goat anti-NeuroD1 (1:500, sc-1084, Santa Cruz Biotechnology, Dallas, TX USA), rabbit anti-phospho-Smad1/5/8 (1:500, AB3848, Millipore), rabbit anti-BDNF (1:500, #47,808, Cell Signaling).

#### Fluorophore-conjugated secondary antibodies (1:250, Thermo Fisher)

Alexa-488 (goat anti-rabbit A11034, goat anti-chicken A-11039, goat anti-rat a11006, goat anti-guinea pig A-11073), Alexa-555 (goat anti-rabbit A-21428, goat anti-mouse A21127), Alexa-647 (goat anti-rat A-21247, goat anti-chicken A21449, goat anti-mouse A21240), and 4,6-diamidino-2-phenylindole (DAPI) was used at 1:1000 for nuclear stain (Invitrogen Hoechst 33,258, Carlsbad, CA, USA).

### Confocal imaging and quantification

Images were acquired using a Leica TCS SP5 Confocal Microscope or Keyence Scanning Microscope. Z-stacks of the DG were obtained (step size: 2 µm) using sequential scanning to prevent bleed-through between fluorophores. Six or more Z-stacks of equal thickness and equivalent rostrocaudal position were quantified for each sample. For per-volume quantification, cells were counted using ImageJ and counts were normalized to the volume of the DG granule cell layer. All imaging and quantification were performed blinded to experimental conditions.

### Statistical analysis

Statistical analyses were performed via two-tailed Student’s *t* test, Pearson’s correlation, two-way Repeated Measures (RM) ANOVA, two-way ANOVA with Šídák’s or Tukey’s post-hoc test for multiple comparisons testing, or three-way ANOVA as indicated in the figures and figure legends. GraphPad Prism 9 software was used for the analyses. Equality of variances was verified using the *F* test of equality of variance. All data are reported as means ± standard error of the mean (s.e.m.). The significance threshold used was *p* < 0.05. The exact sample size for each experimental condition is represented in the figures as symbols and provided in the Supplementary Materials. Complete statistical data for each experiment are also provided in the Supplementary Materials.

### Supplementary Information

Below is the link to the electronic supplementary material.

S1: Analysis of sex differences and dosing schemes. a There were no significant sex differences in our behavioral tests after single or multiple doses of ketamine. b There is a significant difference by three-way ANOVA for the interaction of time (DPI), single or multiple doses (dosing), and ketamine or saline group mice (drug). Supplementary file1 (TIF 48341 KB)

S2: Analysis of CR+, EGR1+, and double-labeled cells/mm3 at 14DPI. Supplementary file2 (TIF 6896 KB)

S3: Analysis of neurogenesis at 1DPI. a Quantification of the total number of cells in the immature lineage, defined by BrdU, DCX, and/or CR positivity, per mm3. Unpaired t-test: t6=8.616 ***p=0.0001. b Fold changes of the number of cells/mm3 labeled by the specified marker(s) relative to saline treatment group. FC=1 indicates no difference from saline. c Representative staining and quantification of NeuroD+, CR+, and double-positive cells in mice given one dose of saline or ketamine 24 hours prior to SC. d Correlation of behavior score and DCX+ cells/mm3 for mice given multiple doses of saline or ketamine 14 days before BT and SC. Pearson R = 0.8673, R2= 0.7522 **p= 0.0053. e Correlations of behavior score and either EGR1+ cells/mm3, percentage of CR+ cells that are CR+EGR1+ double-positive, or percentage of EGR1+ cells that are CR+EGR1+ double-positive, for mice given multiple doses of saline or ketamine 14 days before BT and SC. All ns. Supplementary file3 (TIF 47110 KB)

S4: Analysis of BMP signaling related proteins and BDNF expression in the DG after multiple doses of ketamine, related to Figure 4. a There was no significant correlation between behavior score and pSMAD1/5/8 levels in mice given multiple doses of saline or ketamine, ending 7 days prior to BT and SC. Pearson R = -0.5197 ns p= 0.1869. b There was no significant correlation between behavior score and Noggin levels in mice given multiple doses of saline or ketamine ending 7 days prior to BT and SC. Pearson R ≈ 0 ns p= 0.9983. c There was no significant correlation between behavior score and BMP4 levels in mice given multiple doses of saline or ketamine ending 7 days prior to BT and SC. Pearson R = -0.5473 ns p= 0.1603. d There was no significant correlation between behavior score and BDNF levels in mice given multiple doses of saline or ketamine ending 7 days prior to BT and SC. Pearson R = 0.4765 ns p= 0.2326. e Representative western blots of dentate gyrus tissue probed for total Smad1/5/8 (tSMAD1/5/8) and the loading control GAPDH in mice treated with multiple doses of saline or ketamine. f Quantification of western blots by densitometric analysis of tSMAD1/5/8 in ketamine-treated mice relative to saline-treated mice. Two-way ANOVA: Interaction ** p = 0.0085, DPI ****p <0.0001, ketamine ***p = 0.0006. Šídák's multiple comparisons test: 7DPI S (n=4) v K (n=4) ns p = 0.98, 14DPI S (n=4) v K (n=4) ***p = 0.0006, 21DPI S (n=4) v K (n=4) *p < 0.033. g There was a significant correlation between behavior score and tSMAD1/5/8 levels in mice given multiple doses of saline or ketamine ending 7 days prior to BT and SC. Pearson R = -0.7073 *p=0.497. h There was a significant correlation between behavior score and tSMAD1/5/8 levels in mice given multiple doses of saline or ketamine ending 14 days prior to BT and SC. Pearson R = -0.8424 **p= 0.0087. i There was a significant correlation between behavior score and tSMAD1/5/8 levels in mice given multiple doses of saline or ketamine ending 21 days prior to BT and SC. Pearson R = -0.8674 **p= 0.0053. j Representative IHC staining of pSMAD (red), and DAPI (blue) in the DGs of mice 14 days after cessation of multiple doses of saline or ketamine. Scale bars represent 50um. k Representative IHC staining of BDNF (red), and DAPI (blue) in the DGs of mice 14 days after cessation of multiple doses of saline or ketamine. Scale bars represent 50um. Supplementary file4 (PNG 5864 KB)

S5: Representative IHC staining of mCherry showing the expression of the lentiviruses in the DG. Supplementary file5 (PNG 1637 KB)

Supplementary file6 (PDF 1239 KB)

Supplementary file7 (PDF 489 KB)

## Data Availability

All data needed to evaluate the conclusions in the paper are present in the paper and/or the Supplementary Materials. Supplementary information is available on the journal’s website.
