# Supplementary figures and images for: Ketamine’s rapid and sustained antidepressant effects are driven by distinct mechanisms
Source: Cell Mol Life Sci. 2024 Feb 27;81(1):105. doi: 10.1007/s00018-024-05121-6 (PMC10899278; doi:10.1007/s00018-024-05121-6)

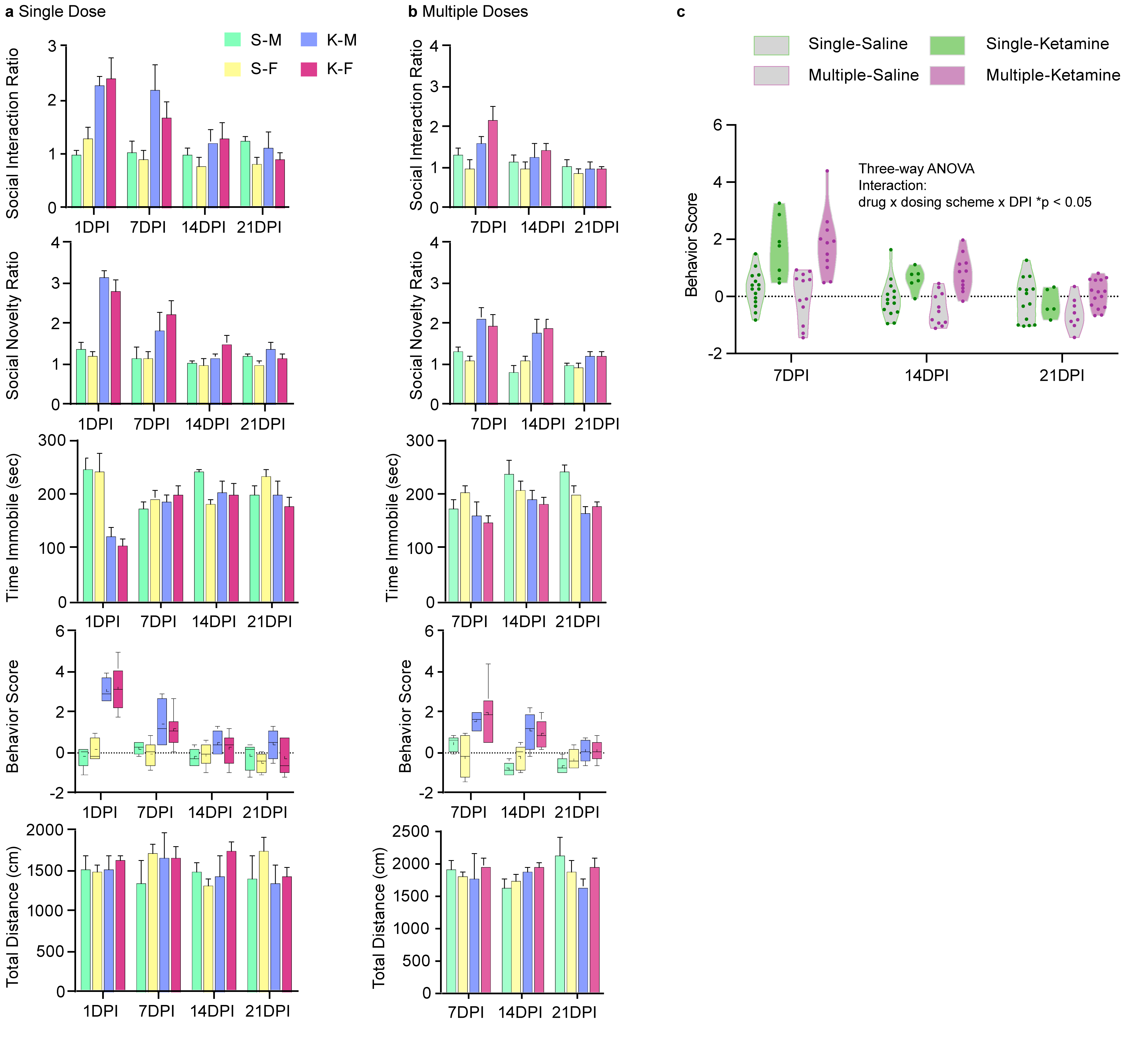

Supplement: Supplementary file 1 — S1: Analysis of sex differences and dosing schemes. a There were no significant sex differences in our behavioral tests after single or multiple doses of ketamine. b There is a significant difference by three-way ANOVA for the interaction of time (DPI), single or multiple doses (dosing), and ketamine or saline group mice (drug). Supplementary file1 (TIF 48341 KB) [file 18_2024_5121_MOESM1_ESM.tif]

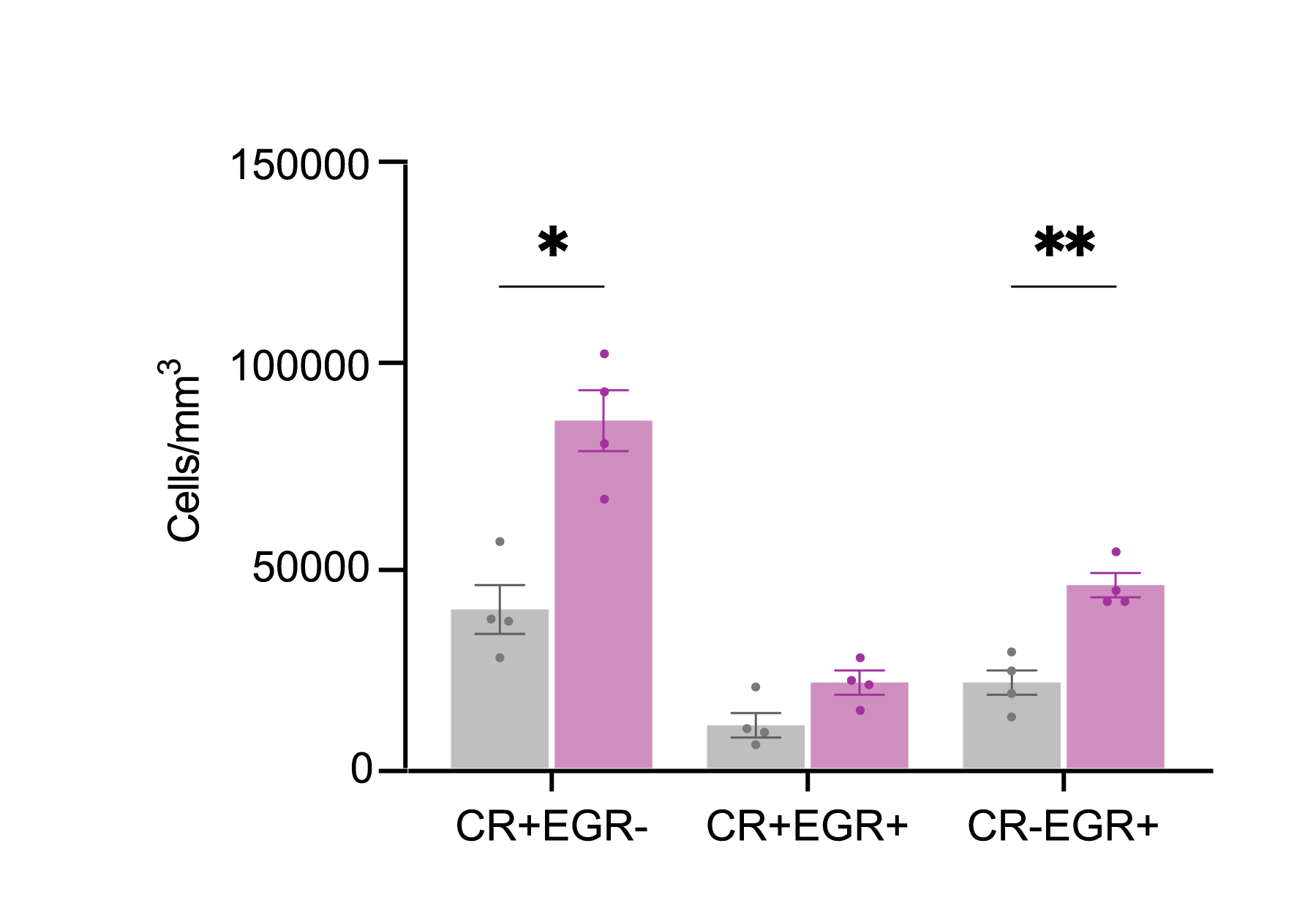

Supplement: Supplementary file 2 — S2: Analysis of CR+, EGR1+, and double-labeled cells/mm3 at 14DPI. Supplementary file2 (TIF 6896 KB) [file 18_2024_5121_MOESM2_ESM.tif]

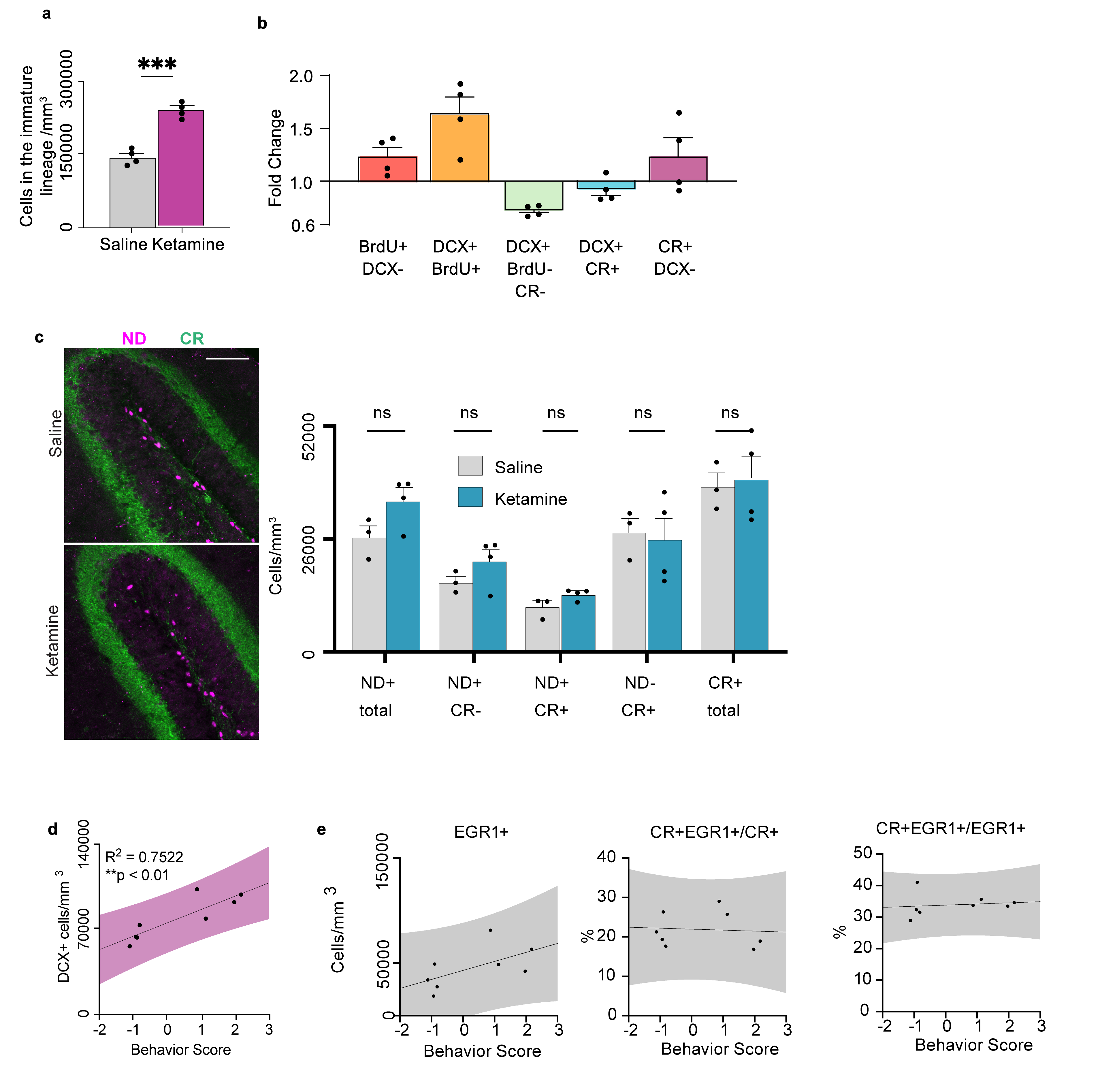

Supplement: Supplementary file 3 — S3: Analysis of neurogenesis at 1DPI. a Quantification of the total number of cells in the immature lineage, defined by BrdU, DCX, and/or CR positivity, per mm3. Unpaired t-test: t6=8.616 ***p=0.0001. b Fold changes of the number of cells/mm3 labeled by the specified marker(s) relative to saline treatment group. FC=1 indicates no difference from saline. c Representative staining and quantification of NeuroD+, CR+, and double-positive cells in mice given one dose of saline or ketamine 24 hours prior to SC. d Correlation of behavior score and DCX+ cells/mm3 for mice given multiple doses of saline or ketamine 14 days before BT and SC. Pearson R = 0.8673, R2= 0.7522 **p= 0.0053. e Correlations of behavior score and either EGR1+ cells/mm3, percentage of CR+ cells that are CR+EGR1+ double-positive, or percentage of EGR1+ cells that are CR+EGR1+ double-positive, for mice given multiple doses of saline or ketamine 14 days before BT and SC. All ns. Supplementary file3 (TIF 47110 KB) [file 18_2024_5121_MOESM3_ESM.tif]

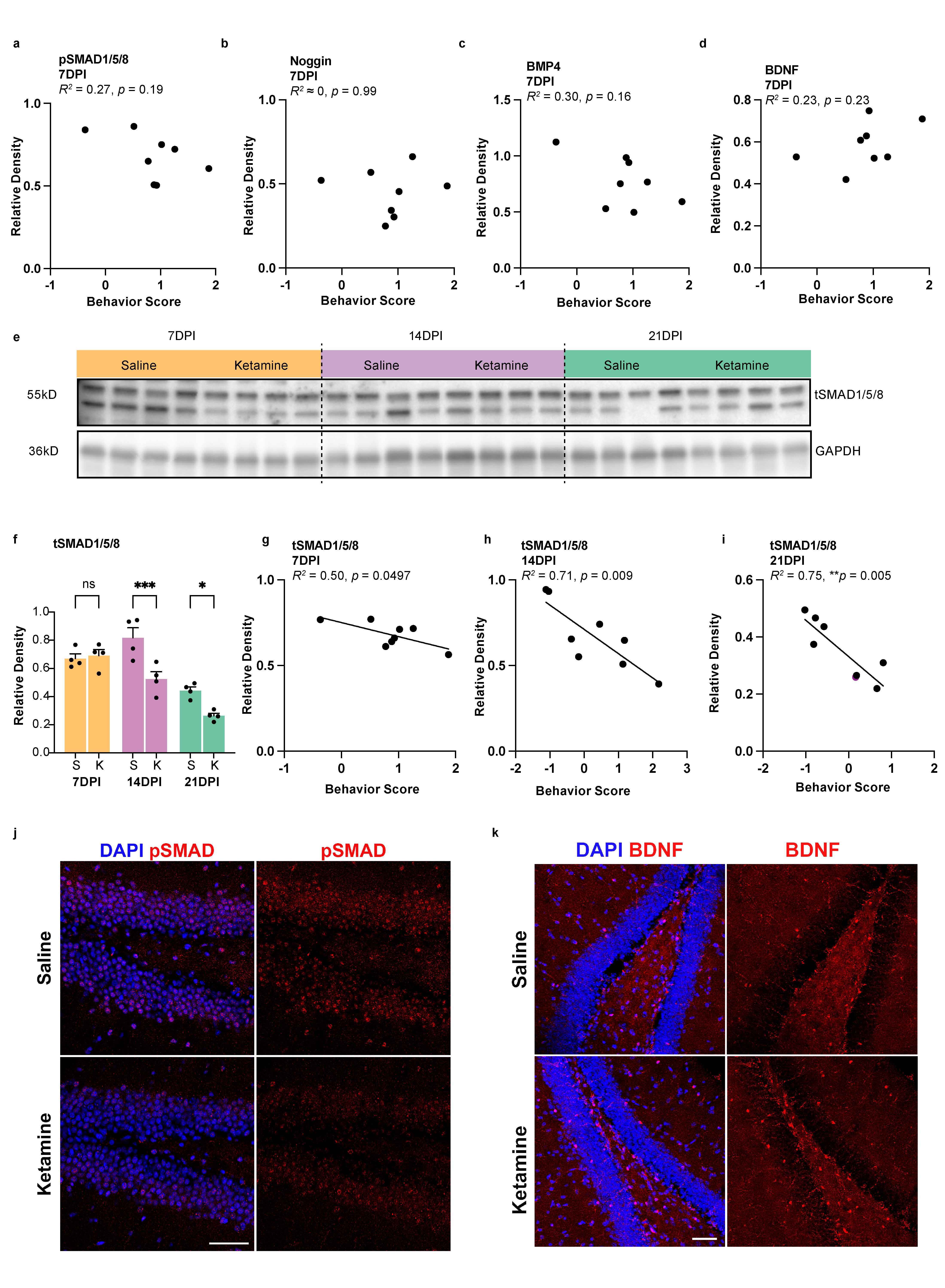

Supplement: Supplementary file 4 — S4: Analysis of BMP signaling related proteins and BDNF expression in the DG after multiple doses of ketamine, related to Figure 4. a There was no significant correlation between behavior score and pSMAD1/5/8 levels in mice given multiple doses of saline or ketamine, ending 7 days prior to BT and SC. Pearson R = -0.5197 ns p= 0.1869. b There was no significant correlation between behavior score and Noggin levels in mice given multiple doses of saline or ketamine ending 7 days prior to BT and SC. Pearson R ≈ 0 ns p= 0.9983. c There was no significant correlation between behavior score and BMP4 levels in mice given multiple doses of saline or ketamine ending 7 days prior to BT and SC. Pearson R = -0.5473 ns p= 0.1603. d There was no significant correlation between behavior score and BDNF levels in mice given multiple doses of saline or ketamine ending 7 days prior to BT and SC. Pearson R = 0.4765 ns p= 0.2326. e Representative western blots of dentate gyrus tissue probed for total Smad1/5/8 (tSMAD1/5/8) and the loading control GAPDH in mice treated with multiple doses of saline or ketamine. f Quantification of western blots by densitometric analysis of tSMAD1/5/8 in ketamine-treated mice relative to saline-treated mice. Two-way ANOVA: Interaction ** p = 0.0085, DPI ****p <0.0001, ketamine ***p = 0.0006. Šídák's multiple comparisons test: 7DPI S (n=4) v K (n=4) ns p = 0.98, 14DPI S (n=4) v K (n=4) ***p = 0.0006, 21DPI S (n=4) v K (n=4) *p < 0.033. g There was a significant correlation between behavior score and tSMAD1/5/8 levels in mice given multiple doses of saline or ketamine ending 7 days prior to BT and SC. Pearson R = -0.7073 *p=0.497. h There was a significant correlation between behavior score and tSMAD1/5/8 levels in mice given multiple doses of saline or ketamine ending 14 days prior to BT and SC. Pearson R = -0.8424 **p= 0.0087. i There was a significant correlation between behavior score and tSMAD1/5/8 levels in mice given multiple doses of saline or ketami [file 18_2024_5121_MOESM4_ESM.png]

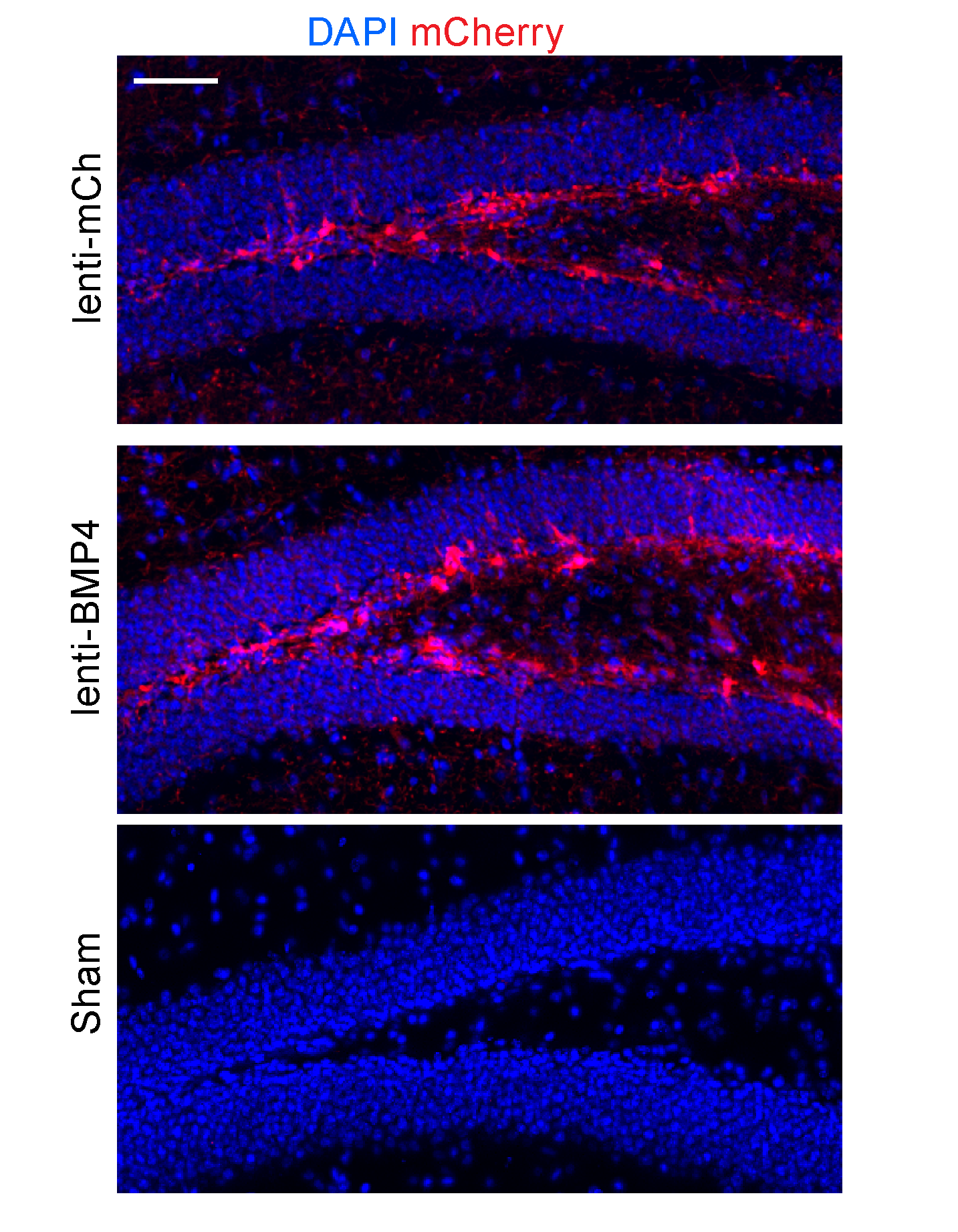

Supplement: Supplementary file 5 — S5: Representative IHC staining of mCherry showing the expression of the lentiviruses in the DG. Supplementary file5 (PNG 1637 KB) [file 18_2024_5121_MOESM5_ESM.png]
